# Single-Cell 5 μm-Resolution Dual-Polarity MALDI-MS Imaging without Matrix Reapplication

**DOI:** 10.1021/acs.analchem.5c03289

**Published:** 2025-07-29

**Authors:** Yanyan Chen, Rui Shi, Jianing Wang, Chengyi Xie, Yuanyuan Song, Ruxin Li, Luyao Wen, Thomas Ka-Yam Lam, Zhu Yang, Zongwei Cai

**Affiliations:** † State Key Laboratory of Environmental and Biological Analysis, 26679 Hong Kong Baptist University, Hong Kong SAR 315200, China; ‡ Department of Chemistry, Hong Kong Baptist University, Hong Kong SAR 315200, China; § School of Marine Science and Engineering, Hainan University, Haikou 570228, China; ∥ Department of Biology, Hong Kong Baptist University, Hong Kong SAR 315200, China; ⊥ Eastern Institute of Technology, Ningbo 315100, China

## Abstract

High-resolution mass spectrometry imaging (MSI) plays a vital role in lipidomics, yet challenges persist in analyzing lipids at the single-cell level due to limitations in spatial resolution and lipid coverage. While existing strategies based on a single matrix application step for dual-polarity provide high lipid coverage from the same sample and enable easy sample preparation, matrix depletion limits their spatial resolution to 10 μm, preventing their application to single-cell imaging. Here, we present a single-cell/subcellular resolution strategy for dual-polarity matrix-assisted laser desorption and ionization mass spectrometry imaging (MALDI-MSI) that eliminates the need for matrix reapplication. This approach achieves 5 μm spatial resolution while maintaining lipid coverage comparable to multistep single-cell imaging methods. This is enabled by a fine-tuned matrix deposition technique that fully utilizes the high sensitivity of *N*-(1-naphthyl)-ethylenediamine dihydrochloride (NEDC) in dual polarities and optimized acquisition conditions, allowing single-deposition workflows without the need for washing, repreparation, or image recalibration. This single-cell resolution MALDI-MSI strategy successfully imaged a broader range of lipid species with distinctive spatial detail in mouse kidney tissue and lung carcinoma cells (A549). Using spatial probabilistic latent semantic analysis (PLSA), we identified three distinct lipid distribution patterns within a single-cell population in both polarities, and histogram analysis revealed substantial cell-to-cell lipidomic heterogeneity. This strategy overcomes limitations of traditional dual-polarity MSI and provides a powerful tool for advancing cellular lipidomics, elucidating disease mechanisms, and investigating environmental toxicology.

## Graphical abstract

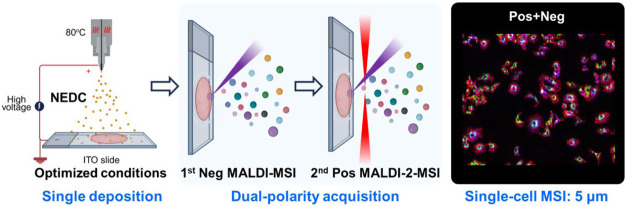

## Introduction

Matrix-assisted laser desorption/ionization-mass spectrometry imaging (MALDI-MSI) is a powerful technique for the spatially resolved biomolecular analysis, offering unique insights into the distribution and composition of lipids in tissue sections.^1,2^ Lipids are essential components of cellular structures^3^ and key mediators in signaling and energy metabolism,^4,5^ making their spatial profiling crucial for understanding health, disease mechanisms, and environmental effects.^6–9^ For example, Sun *et al.* have utilized MALDI-MSI to characterize lipid heterogeneity within gastric cancer tissues, revealing insights into disease pathology.^10^ Despite its impressive capabilities, the application of MALDI-MSI at the cellular level remains challenging, such as limited spatial resolution, reduced sensitivity, and insufficient lipid coverage. Conventional MALDI methods typically operate with pixel sizes between 20 to 50 μm, which exceed the dimensions of individual mammalian cells.^11^ Consequently, the lipid profiles obtained represent an average over multiple cells, potentially masking critical details of cellular lipid metabolism and diluting the data specificity. Achieving cellular-level resolution in MALDI-MSI is thus essential for uncovering lipidomic heterogeneity and advancing our understanding of lipid function in cellular processes.^12–15^

Achieving single-cell resolution in MALDI-MSI remains a challenge due to the trade-off between spatial resolution and ionization sensitivity. Previous studies have indicated that the fraction of molecules effectively ionized is typically less than 0.1% of the total analyte present in the initial sample,^16,17^ resulting in mass spectra dominated by highly abundant or easily ionized molecules while underrepresenting less abundant or less efficiently ionized species, potentially masking biologically relevant information.^18^ These issues are further amplified in high-resolution imaging, particularly when achieving spatial resolutions of 10 μm or less, where reduced sample volumes per pixel diminish sensitivity and hinder the detection of low-abundance molecules, limiting the comprehensive analysis of complex biological samples.^19^ To address these sensitivity issues, the recent development of a laser-based postionization method, known as MALDI-2, represents a significant breakthrough.^15,20^ MALDI-2 has been reported to increase the ion yield by two or three orders of magnitude in both ionization modes for various analyte classes, including phospholipids (PL), neutral lipids, and metabolites.^21–23^ This advancement holds great promise for advancing high-resolution and single-cell lipidomics.

Despite these advances, sample preparation methods remain critical in achieving subcellular high-resolution MSI while maintaining high coverage, particularly where spatial resolutions of 5 μm are required. The selection and deposition of the matrix play essential roles in MALDI-MSI analysis.^24,25^ For high-resolution imaging, sublimation is often used because it produces smaller matrix crystals;^26,27^ however, this method suffers from low extraction efficiency, resulting in fewer detectable peaks. Recrystallization processes, such as heat treatment or interaction with solvent vapor, have been introduced to address this issue.^28,29^ Conversely, pneumatic-assisted deposition methods can improve extraction efficiency but may cause analyte diffusion.^14^ These challenges can lead to weak signals, fewer detected peaks, and low molecular coverage, undermining the potential of high-resolution MSI. On other hand, dual-polarity imaging is critical for comprehensive lipid profiling, as certain lipid classes preferentially ionize in either positive or negative ionization mode.^30,31^ For dual-polarity MALDI-MSI, the common workflow is to perform each polarity on adjacent tissue sections or cell slides using the optimized matrix. Another approach is to acquire both polarities from the same area of tissue or cells. Strategies using a single matrix application step simplify this process by enabling the acquisition of both polarities from a single sample without matrix reapplication. However, achieving high spatial resolution (<10 μm) with these strategies remains challenging due to matrix depletion caused by the laser spot fully overlapping with the sample area, which compromises signal quality and reproducibility. In contrast, prior studies achieving resolutions to 10 μm and above utilized the sparse scanning of laser spot (5 μm in diameter) to mitigate depletion effects, this approach fully exploits the instrument’s highest resolution potential and limits its applicability for single-cell imaging.^26,30^ Consequently, conventional workflows generally involve recoating or washing and reapplying the matrix to facilitate dual-polarity acquisition at high spatial resolutions.^14,32^ This increases labor while potentially causing lipid diffusion, signal loss, and misalignment between images. Therefore, developing an optimized matrix deposition method is crucial to balancing crystal size, extraction efficiency, and spatial integrity, ultimately maximizing the quality of high-resolution MSI results. Our extensive research has demonstrated the exceptional performance of the *N*-(1-naphthyl)-ethylenediamine dihydrochloride (NEDC) matrix in detecting and imaging a variety of lipids in negative ionization mode, providing high-quality images with high spatial resolution within tissue sections.^33,34^ Furthermore, our previous study revealed that NEDC, when combined with the MALDI-2 function, also performs remarkably well in positive ionization mode, enabling the effective detection of metabolites and yielding data quality comparable to that achieved in negative ionization mode.^35^

In this study, we present a single-cell/subcellular-level MALDI-MSI strategy that employs a single deposition step without requiring additional matrix reapplication, addressing the challenges of traditional dual-polarity MALDI-MSI analysis and achieving high coverage spatial lipidomics. For the first time, this approach enabled repeated dual-polarity mass spectrometry imaging with full laser spot coverage of the sample. By integrating the optimized sample preparation and dual-polarity matrix NEDC with the optimized acquisition strategies, we achieved 5 μm spatial resolution while maintaining lipid coverage comparable to multistep single-cell imaging methods.

## Materials and Methods

### Chemicals and Reagents

*N*-(1-naphthyl)-ethylenediamine dihydrochloride (NEDC, ≥99%), 2,5-dihydroxybenzoic acid (DHB, ≥98%), 9-aminoacridine (9AA, ≥99.5%), and ammonium formate were obtained from Sigma-Aldrich (St. Louis, MO). HPLC grade methanol (MeOH) was obtained from VWR International Company (Radnor, Pennsylvania). Dulbecco’s Modified Eagle’s Medium (DMEM), fetal bovine serum (FBS), penicillin-streptomycin, 0.25% trypsin-ethylenediaminetetraacetic acid (trypsin-EDTA), and phosphate-buffered saline (PBS) were obtained from Thermo Fisher (Cambridge, MA, USA).

### Animal Experiments

The six-week-old male C57BL/6 mice were obtained from the Laboratory Animal Service Centre of the Chinese University of Hong Kong and housed in a controlled environment. They were kept in a room with the 12 h light cycle, maintained at a temperature of 22 ± 2 °C and a relative humidity of 45%. The mice had free access to food and water throughout the duration of the study. All animal experiments conducted in this study followed the guidelines of the use of experimental animals of HKBU and were approved by the HKBU Committee on the Use of Human & Animal Subjects in Teaching and Research.

### Tissue Preparation

Mouse kidneys were embedded in iced saline solution and subsequently sliced into 10 μm thick sections using a CryoStar Nx70 cryostat (Thermo Fisher Scientific, Walldorf, Germany) at −20 °C. These tissue sections were then carefully mounted onto the indium tin oxide (ITO) conductive glass slides and placed in a vacuum desiccator for 30 min prior to matrix coating.

### Cell Experiments

Lung carcinoma cells (A549) were obtained from the American Type Culture Collection (ATCC, Manassas, VA). A549 cells were grown in a 10 cm dish in 8 mL DMEM with 10% FBS and 1% penicillin-streptomycin in a humidified atmosphere at 37 °C with 5% CO_2_.

In cell preparation for the imaging experiment, we followed a previous established protocol with slight modifications.^27^ Initially, cells were cultured directly on ITO conductive glass slides. The clean ITO slide was placed in the 10 cm dish, and approximately 1 × 10^4^ cells were seeded into the dish incubating for overnight. The medium was removed from the slide by washing it twice with iced 150 mM ammonium formate after incubation. Then, the cells were fixed in iced 4% formaldehyde solution for 5 min at room temperature, followed by another two washed with iced 150 mM ammonium formate. Cells were subsequently placed in a vacuum desiccator for 30 min to facilitate the imaging experiment. Three ITO slides were prepared as replicates.

### Matrix Coating and Microscopy

Following the drying process, tissue sections or cells were coated with matrix using our home-built electron-thermo-spray device (Figure S1). The matrix application process was carefully optimized for each matrix to achieve optimal results (Details in the Supporting Information). For NEDC, a 5 mg/mL solution was prepared in a MeOH/H_2_O (*v*/*v*, 7:3) solvent mixture. The spray parameters for NEDC were fine-tuned to achieve optimal results: a flow rate of 10 μL/min, nitrogen flow pressure set at 80 psi, and nozzle temperature maintained at 80 °C to reduce drying time and minimize crystal size. The distance between the sample surface and the nozzle was established at 3 cm with a spraying voltage of 5000 V and an XY velocity of 1098 mm/min. To ensure optimal coverage, ten layers of NEDC were applied. In contrast, DHB (20 mg/mL in MeOH) and 9AA (5 mg/mL in MeOH) were sprayed at a lower nozzle temperature of 60 °C, requiring 12 and 10 layers, respectively. All other spraying parameters remained consistent across the different matrices. The matrix particle size was measured by scanning electron microscopy (SEM, Crossbeam 350, ZEISS, Germany). For SEM analysis, the matrix was sprayed directly onto the ITO slide.

### MALDI-MSI Measurement

The MSI measurement was performed using a timsTOF fleX MALDI-2 (Bruker Daltonics, US) equipped with microGRID and SmartBeam 3D laser (355 nm wavelength). MALDI-MS spectra were obtained at the mass range from *m*/*z* 150 to 2000 in both positive and negative ionization modes. Following the acquisition of data in negative ionization mode using MALDI, the instrument was switched to positive ionization mode, and data were acquired using the MALDI2 laser over the same area. For MALDI imaging at a spatial resolution of 10 μm, negative ionization mode (MALDI) spectra were acquired by using 10 laser shots per pixel at a laser power of 60%, whereas positive ionization mode (MALDI-2) spectra were acquired by using 10 laser shots per pixel at a laser power of 90%. At a spatial resolution of 5 μm, negative ionization mode (MALDI) spectra were acquired using 8 laser shots per pixel at a laser power of 70%, while positive ionization mode (MALDI-2) spectra were acquired using 10 laser shots per pixel at a laser power of 95%. The laser repetition rates were set to 10,000 Hz for MALDI and 1000 Hz for MALDI-2. For new lasers with low cumulative shot counts, the pulse energy and shot number must be appropriately lowered. The MS spectra were obtained directly on tissue or cell sections using the corresponding laser spot size. Data acquisition was conducted with TimsControl (Bruker Daltonics). We used Agilent ESI-L low-concentration tuning mixes to calibrate the MALDI instrument.

### MSI Data Analysis

MALDI-Q-TOF MS and MS/MS data were conducted by DataAnalysis (Version 5.3, Bruker Daltonics, Germany). The MS spectra were obtained to identify endogenous lipids by comparison with LIPID MAPS within a mass error of less than 5 ppm. [M + H]^+^, [M + Na]^+^, [M + K]^+^, and [M – H_2_O + H]^+^ were detected in positive ionization mode, and [M – H]^−^ was analyzed in negative ionization mode. The MS/MS analysis was performed directly in tissue sections. The spatial images of endogenous metabolites were analyzed by a SCiLS Lab 2022b (Bruker Daltonics, Germany). Root mean square (RMS) was selected to normalize the MSI data. Dimensionality reduction and clustering of single-cell data were performed using the probabilistic latent semantic analysis (PLSA) function in the SCiLS Lab. For histogram analysis, image segmentation was conducted in SCiLS Lab to identify individual cells, followed by manual selection of each cell to extract the corresponding mass spectral peaks for further analysis. ImageJ was used to merge images from different ion channels.

## Results and Discussion

### NEDC as a Superior Dual-Polarity Matrix for Lipid Detection

The development of robust methodologies for dual-polarity lipid detection is essential to advancing lipidomics, particularly for high-spatial-resolution imaging at the cellular level. To this end, we evaluated the suitability of NEDC as a dual-polarity lipid detection matrix, and we first compared the NEDC matrix with other commonly used matrix, namely DHB and 9AA, in traditional MALDI-MSI and MALDI-2-MSI at a standard 50 μm spatial resolution. In positive ionization mode, MALDI-2 significantly enhanced lipid detection compared to traditional MALDI (Figure S2A), yielding nearly a five-fold increase in imaged lipid species (394 vs 80) when using NEDC as the matrix (Figure S3A). The method expanded the range of imaged lipid classes, including GPs, glycerolipids (GLs), and sphingolipids (SPs), while also improving visualization of neutral lipids, such as diacylglycerols (DGs) and triacylglycerols (TGs) (Figure S3C). The results found that the use of the NEDC matrix outperformed the commonly used DHB matrix in positive ionization mode, particularly in the observation of PE and SHexCer (Figure S3C and E). In negative ionization mode, the performance of the NEDC matrix was comparable in both MALDI and MALDI-2 (Figure S2B), with 248 and 253 imaged lipids, respectively (Figure S3B). In addition, the use of the 9AA matrix, traditionally favored in negative ionization mode, showed a similar phenomenon (little enhancement) as the NEDC matrix in MALDI-2-MSI analysis. It was found that the range and amount of lipids imaged using 9AA as matrix were nearly fully covered by the use of NEDC as a matrix (Figure S3B, D and F). The superior enhancement effect of NEDC in MALDI-2, particularly in positive ionization mode, may be attributed to the presence of HCl in the NEDC matrix, which likely promotes the formation of more clusters during the initial MALDI process. Under MALDI-2 conditions, these abundant clusters can be further ionized, leading to improved ionization efficiency and broader coverage of the lipid. The lesser enhancement in negative ionization mode might be explained by NEDC already exhibiting high ionization efficiency under traditional MALDI, thereby reducing the impact of MALDI-2. These findings highlight the synergistic effect between NEDC and MALDI-2 technology, particularly in positive ionization mode, offering a powerful approach to comprehensive lipid profiling.

Having confirmed that NEDC is suitable for dual-polarity analysis, we next evaluated its performance in high-resolution imaging. Matrix crystal size critically influences spatial resolution due to laser focusing limitations and pixel size requirements.^24^ Traditional methods, such as pneumatic-assisted deposition, achieve high ionization efficiency but risk signal diffusion. While sublimation produces nanoscale crystals that enhance spatial resolution, achieving high sensitivity with this approach often requires recrystallization processes, such as heat treatment or exposure to solvent vapor, to improve ion yield.^26–29^ In our study, pneumatic spray deposition was employed to ensure sufficient sensitivity, while the matrix coating process was further optimized using a home-built electron-thermo-spray device to achieve smaller crystal sizes and improved spatial resolution. The resulting crystals of the NEDC matrix were 0.2 × 1 μm in size (Figure S4A), which is small enough for high-resolution MS imaging. Similarly, under optimized conditions, DHB matrix also produced crystals of <1 μm (Figure S4B). To evaluate the performance of NEDC for high-resolution imaging, we tested its lipid detection capacity on mouse kidney sections at a lateral resolution of 10 μm, comparing it with DHB. The spectral results found that they exhibited similar molecular profiles between these two matrixes (Figure S4C). Specifically, NEDC allowed the visualization of 319 different lipid species, whereas the use of DHB as a matrix enabled the observation of 283 lipid species (Figure S4D) within tissue sections. We observed that six specific lipid species associated with glomeruli, including [Cer 34:2;O+H]^+^, [Cer 36:5;O+H]^+^, [PE 32:4+H]^+^, [CerP 36:2;O_2_+K]^+^, [SM 34:1;O_2_+H]^+^, and [SM 34:0;O_2_+H]^+^ were identified using NEDC as the matrix (Figure S5). [Cer 34:2;O+H]^+^ and [CerP 36:2;O_2_+K]^+^ can be imaged by the use of both matrix (Figure S5A and B). However, for [Cer 36:5;O+H]^+^ and [SM 34:1;O_2_+H]^+^, the signals were insufficient to yield a high-quality image using DHB as the matrix (Figure S5C and D). In addition, [PE 32:4+H]^+^ and [SM 34:0;O_2_+H]^+^ were not detectable when using DHB as the matrix (Figure S5E and F).

In addition to enabling the detection of more lipid species, NEDC provided superior spatial resolution and sharper images in the mouse kidney sections. As shown in Figure S4E and F, NEDC achieved more precise spatial distributions of detected lipids compared to DHB. Besides, the use of NEDC as the matrix also showed good image quality with clear visualization of glomeruli ([CerP34:1;O_2_–H]^−^ (orange)) and other regions (Figure S6B) in negative ionization mode. NEDC achieves superior imaging quality and coverage compared to DHB in positive mode at MALDI-2, while also being well-established for its excellent performance in high-resolution lipid imaging in negative ion mode. Therefore, we selected NEDC as the matrix for the single-cell/subcellular-level MALDI-MSI strategy, which enables dual-polarity imaging without requiring matrix removal or reapplication.

### Dual-Polarity MALDI-MSI Lipid Profiling without Matrix Reapplication

Building on the above results, we next explored the potential of using the NEDC matrix to simultaneously acquire positive and negative ionization mode data on the same kidney section. In this approach, we first collected data in the negative ionization mode using MALDI. Since negative ionization with the NEDC matrix requires lower laser energy, the matrix was largely preserved, enabling effective subsequent imaging in the positive ionization mode using MALDI-2 (Figure 1A). We then compared the spectra obtained from a single matrix application step for the dual-polarity MALDI-MSI strategy with those acquired from a single acquisition. The results showed that the double acquisition of this method produced spatial patterns that closely matched those from the single acquisition, with similar lipid profiles (Figure S7A and B). The identified lipid species were summarized in the Tables S1 and S2. As shown in Figure 1B, [CerPE 36:1–H]^−^ at *m*/*z* 687.546 and [SHexCer 40:1;O_3_–H]^−^ at *m*/*z* 878.603 were imaged in the first round under negative ionization mode, while [SM 33:3; O_5_+K]^+^ at *m*/*z* 771.474 and [PC 32:0+H]^+^ at *m*/*z* 734.569 were captured in the second round with positive ionization mode. As shown in the merged image, distinct renal structures such as the glomeruli (green), cortex (blue), medulla (red and purple), and tubules (red) were clearly visualized, demonstrating the capability of this strategy to provide high-resolution spatial information.

**Figure 1:**
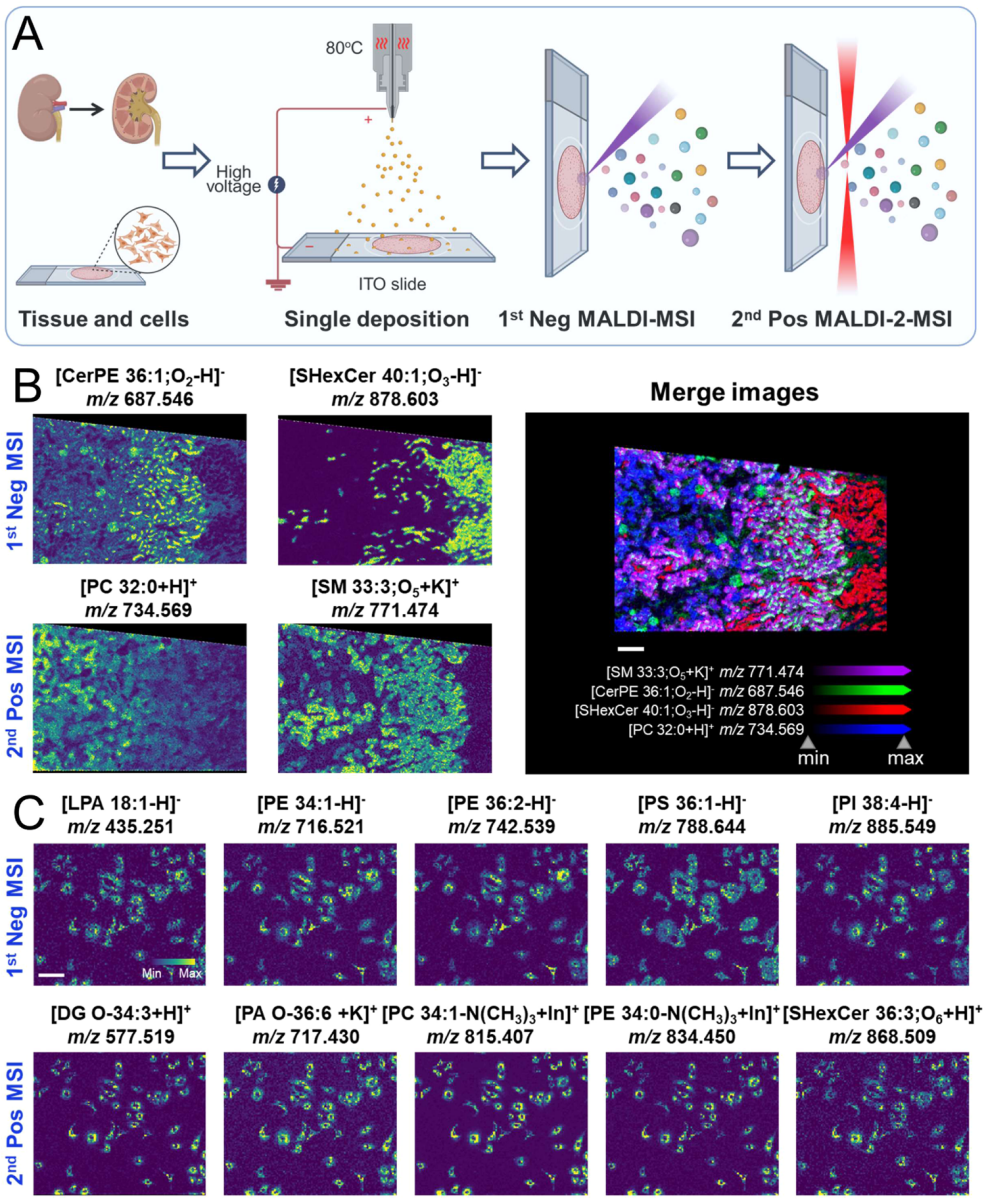
Single-cell resolution images obtained from the same mouse kidney section and A549 cells in dual-polarity modes using MALDI-MSI with a single matrix application step. (A) Schematic diagram illustrating the dual-polarity MALDI-MSI strategy with a single matrix application step. (B) High-resolution imaging of a mouse kidney section at 10 μm spatial resolution. (C) Subcellular resolution imaging of A549 cells at 5 μm spatial resolution.

We further applied this method to single-cell analysis, demonstrating its capability to collect both positive and negative ionization mode data with a single matrix deposition. The in situ MS spectra (Figure S8A) and images (Figure 1C) obtained from the A549 cells showed a variety of lipid species within individual cells in both polarities, revealing complex lipid profiles at the subcellular level. It is noteworthy that numerous PLs were detected as [M+In]^+^ ions in positive ionization mode, which is consistent with a previous report employing transmission-mode MALDI-2-MSI for single-cell analysis.^15^ Interestingly, the formation of [M+In]^+^ ions was not observed in conventional MALDI-MSI (Figure S9), suggesting that this phenomenon might be attributed to the highly focused laser beam employed in the MALDI-2 ablation process on the ITO surface. Furthermore, we investigated the spatial distributions of lipids with different adducts, including [M + H]^+^, [M + In]^+^, and [M + 2In – H]^+^. For example, we found that PC 34:1 and PE 38:4 with these adducts have consistent spatial patterns within cells (Figure S10). The [M + In]^+^ ions displayed enhanced signal intensities relative to those of the [M + H]^+^ counterparts (Figure S10), potentially due to the unique ionization processes facilitated by the MALDI-2 approach. The subsequent MS/MS measurements were conducted to identify the lipids (Figure S11). Approximately one hundred lipid species were tentatively assigned in both ionization modes within cells (Tables S3 and S4). To further evaluate the reliability of our established method, we compared the number of lipid peaks tentatively identified using this approach with those obtained from a single acquisition, where both polarities were collected using MALDI-2. Our results demonstrate that the number of lipid peaks identified with our method is comparable to those obtained from a single acquisition in both kidney tissue (Figure S6E) and A549 cells (Figure S6F). In addition, the image quality of kidney tissue and cells is comparable to that of single acquisitions (Figure S6A–D). This consistency highlights the robustness of MALDI-MSI with a single matrix application step, showing that it can achieve high-quality data acquisition in dual polarities without a significant loss of detected lipid species.

### Decoding Spatial Characteristics within the Cell Population

Decoding the spatial characteristics within cell populations is essential for understanding cellular heterogeneity, as previous studies have demonstrated that even in the same cell population, molecular distributions can vary significantly.^12,13^ To investigate the spatial organization of lipids at the cellular level, we operated dual-polarity high-spatial-resolution imaging without matrix reapplication. By combining positive and negative ionization data acquisition, this approach provided a comprehensive view of lipid distributions within individual cells, offering deep insights into cellular heterogeneity.

To elucidate the spatial distribution patterns of molecular species within individual cells, we performed unsupervised statistical analysis based on the intensities of tentatively assigned *m*/*z* values of lipids at each pixel in both ionization modes. The probabilistic latent semantic analysis (PLSA) allowed us to decompose the lipid profiles into five principal components (*Com*), unveiling distinctive spatial characteristics of lipids within cells (Figure 2 and Figures S12, S13). Compared with the bright-field microscopic image, component 1 (*Com* *#*1) consistently displayed a perinuclear-to-peripheral gradient in both ionization modes (Figure 2B and Figure S13A), *Com* *#*3 was indicative of high expression of molecules at the edge of cytoplasm (Figure 2C and Figure S13B), while *Com* *#*4 (Positive) and *Com* *#*5 (Negative) were distinctive in its ability to detect lipid signals specifically in cell membranes, in addition to other regions (Figure 2D and Figure S13C). Representative lipids from each component further illustrated these spatial patterns. For example, [Hex2Cer 30:6;O_6_+H]^+^ at *m*/*z* 860.464 (*Com* *#*1) exhibited a shift from intracellular regions toward the cell periphery (Figure 2F), while [PA 34:3+K]^+^ at *m*/*z* 709.428 (*Com* *#*3) was also enriched in the cytoplasm edge (Figure 2G). In addition, [LPS 26:2+H]^+^ at *m*/*z* 634.403 (*Com* *#*4), was specifically localized to cell membranes apart from the other two distribution patterns (Figure 2H). Such spatial distributions were further validated in negative ionization mode, where similar patterns were observed for [PE 36:1-H]^−^, [PA 34:1-H]^−^, and [PS 34:1-H]^−^ (Figure S13). Consistent with the Capolupo*et al.*’s observation of a perinuclear-to-peripheral gradient in human dermal fibroblasts;^36^ our analysis also revealed a similar spatial distribution pattern associated with *Com* *#*1. The visualization of two additional distinct spatial patterns (*Com* *#*3 and 4) provides complementary insights into the spatial regulation of molecular networks within cells. In addition to spatial patterns, cell-to-cell variability was evident in the expression levels of certain lipid species. For example, [PS 34:1-H]^−^ displayed a high abundance of some cells while being barely detectable in others (Figure S13H). This phenomenon might contribute to the cell-to-cell heterogeneity in lipid expression, which was similar to previous observations in human dermal fibroblasts.^36^ This approach demonstrates the power of our dual-polarity MALDI-MSI strategy with a single matrix application step in analyzing molecular heterogeneity at subcellular scales, providing a foundation for further understanding the spatial regulation mechanisms that govern cellular processes.

**Figure 2:**
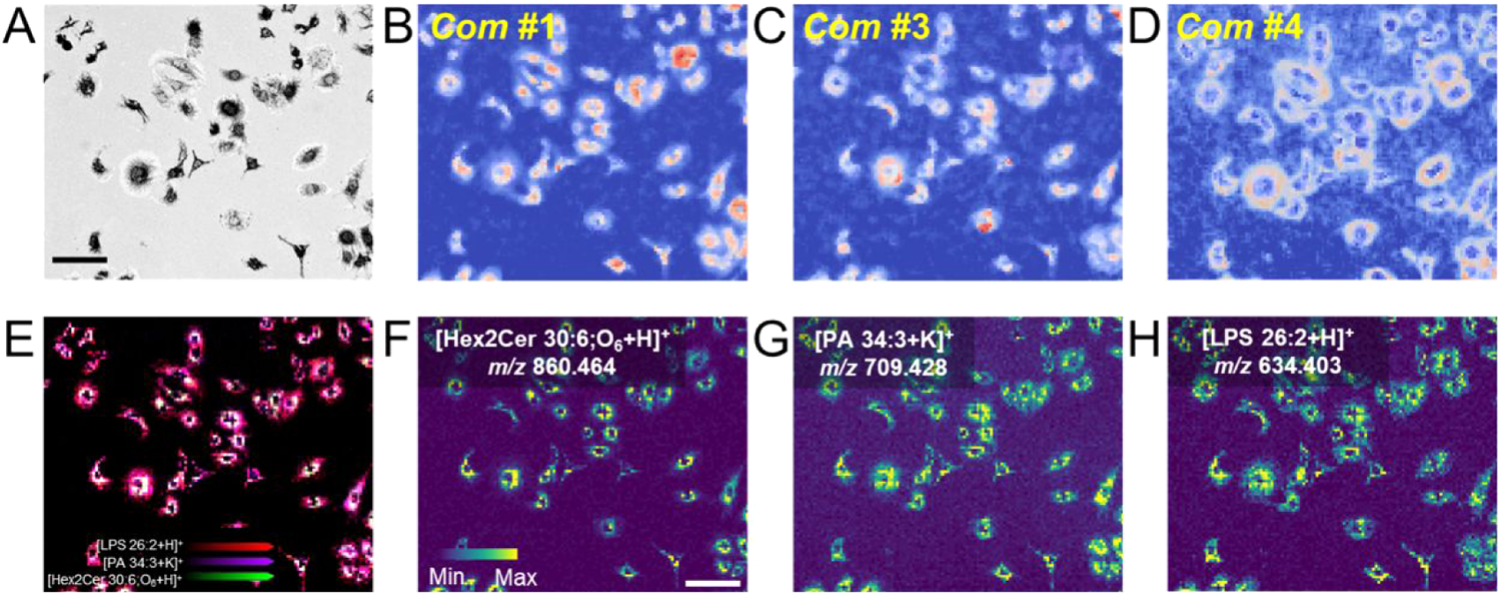
Spatially resolved lipidomics of A549 cells in positive ionization mode. (A) Bright-field microscopy image of A549 cells. (B-D) Lipid-driven in situ probabilistic latent semantic analysis (PLSA) result. (E) Merged lateral resolved signal intensity of [Hex2Cer 30:6;O_6_+H]^+^ (green), [PA 34:3+K]^+^ (purple) and [LPS 26:2+H]^+^ (red). MS images of representative lipids including (F) [Hex2Cer 30:6;O_6_+H]^+^, (G) [PA 34:3+K]^+^ and (H) [LPS 26:2+H]^+^. The scale bar represents 100 μm.

### Cell-to-Cell Heterogeneity in Lipid Distribution

Beyond spatial distribution patterns identified within the cell population, we further investigate the observed cell-to-cell heterogeneity in the lipid expression level. We extracted the signal intensities of 120 cells and represented their signal distributions in the form of histograms. These histograms facilitated visualization of the signal distribution features among cells by categorizing them into bins based on their signal intensities. As a result, histograms with narrow and Gaussian-shaped signal intensity distributions indicated similar signal intensities generated by each cell (Figure 3A), as illustrated by [PC 34:3-N­(CH_3_)_3_+In]^+^ at *m*/*z* 811.377, indicating homogeneous distribution (Figure 3B). Conversely, the histogram featuring broader peaks with wide distribution suggested heterogeneity within cells, for example, [PC 34:2-N­(CH_3_)_3_+In]^+^ at *m*/*z* 813.393 in A549 cells (Figure 3C and D). It should be noted that [PC 34:3-N­(CH_3_)_3_+In]^+^ and [PC 34:2-N­(CH_3_)_3_+In]^+^ belong to the same lipid class, but different species showed distinct spatial features within cells, suggesting that even closely related lipid species can display varying degrees of intercellular heterogeneity. Such spatial variation may reflect specific cellular processes, including lipid synthesis, signaling pathways, or interactions with the cellular microenvironment.^13^ In addition, the histogram of [PE 38:4]^−^ at *m*/*z* 766.537 demonstrated heterogeneity within cells (Figure 3E), with some cells exhibiting high signal intensities while others showed lower signals (Figure 3F). Exploring cellular heterogeneity at the single-cell level provides insights into the complexity and diversity within cell populations. Understanding these variations can enhance our comprehension of cellular behavior, response to external stimuli, and potential implications for disease states associated with lipid dysregulation.

**Figure 3:**
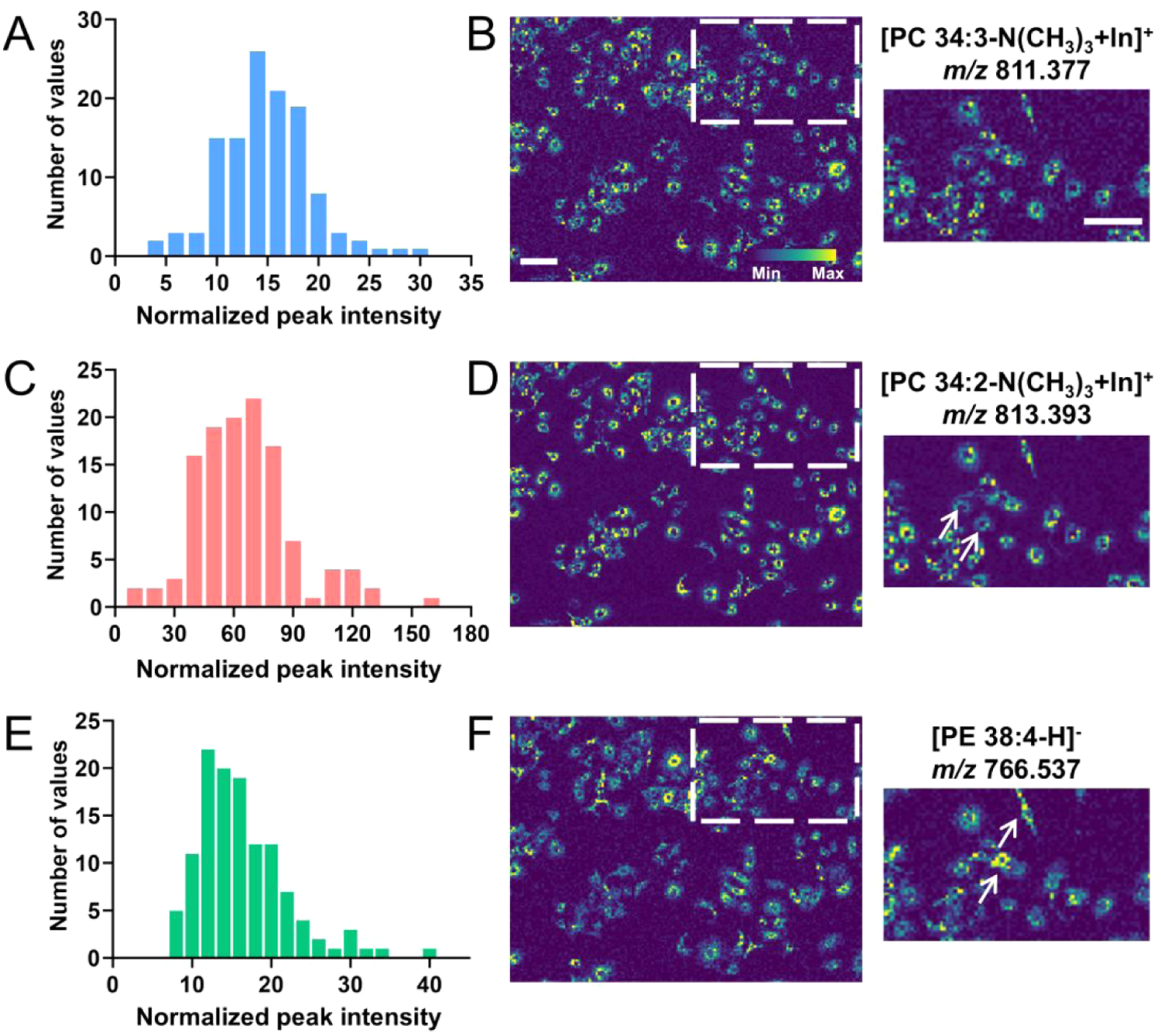
MALDI2-MSI and histograms reveal cell-to-cell heterogeneity. Histograms of (A) [PC 34:3-N­(CH_3_)_3_+In]^+^, (C) [PC 34:2-N­(CH_3_)_3_+In]^+^ and (E) [PE 38:4-H]^−^. MS images of (B) [PC 34:3-N­(CH_3_)_3_+In]^+^, (D) [PC 34:2-N­(CH_3_)_3_+In]^+^, and (F) [PE 38:4-H]^−^, and accompanied zoom-in images. The white arrows indicate the cells with heterogeneous signal intensities. The scale bar represents 100 μm.

## Conclusions

In summary, our study introduces an approach that combines the single-deposition dual-polarity capabilities of the NEDC matrix with MALDI-2-MSI, enabling expanded lipid coverage and high-resolution imaging in both tissues and single cells. Without the need for reapplication of the matrix, we achieved subcellular resolution at 5 μm while maintaining lipid coverage comparable to multistep methods, simplifying sample preparation and preserving the integrity of endogenous molecules for consistent imaging quality. This workflow allowed us to visualize the detailed structure of mouse kidney tissue and single cells, identifying thousands of lipid species with precise spatial localization in both ionization modes. Beyond technical advancements, the strategy uncovered molecular heterogeneity at the single-cell level, revealing distinct lipid distribution patterns and cell-to-cell variability through spatial PLSA. These findings emphasize functional compartmentalization and metabolic diversity within cellular populations, highlighting the critical role of lipids in tissue organization and cellular adaptation. Mapping lipidomic heterogeneity at subcellular resolution opens new opportunities for studying disease mechanisms, cellular stress responses, and metabolic dysregulation, paving the way for future advances in precision medicine and biomarker discovery.

## Supplementary Material



## Notes

List of putative lipids, matrix deposition device, matrix deposition conditions, additional information on comparative results using different matrixes, MSI resutles showing lipid profiles from kidney sections and single cells (PDF)
