# Supplementary material for: Single-Cell 5 μm-Resolution Dual-Polarity MALDI-MS Imaging without Matrix Reapplication
Source: Anal Chem. 2025 Jul 29;97(31):17167–74. doi: 10.1021/acs.analchem.5c03289 (PMC12355473; doi:10.1021/acs.analchem.5c03289)
Supplement: Supplementary file 1 [file ac5c03289_si_001.pdf]

Supporting Information for

Single-Cell 5 μm-Resolution Dual-Polarity MALDI-MS Imaging without Matrix Reapplication

Yanyan Chen[a,b], Rui Shi[a], Jianing Wang[a,c]*, Chengyi Xie[a,b], Yuanyuan Song[a], Ruxin Li[a], Luyao Wen[a], Thomas Ka-Yam Lam[a,b] Zhu Yang [a,d], Zongwei Cai[a,b,e]*

[a] State Key Laboratory of Environmental and Biological Analysis, Hong Kong Baptist University, Hong Kong SAR, China

[b] Department of Chemistry, Hong Kong Baptist University, Hong Kong SAR, China

[c] School of Marine Science and Engineering, Hainan University, Haikou 570228, China

[d] Department of Biology, Hong Kong Baptist University, Hong Kong SAR, China

[e] Eastern Institute of Technology, Ningbo 315100, China

* Corresponding Authors

Jianing Wang, Email: justin.wang.4in1@gmail.com

Zongwei Cai, Email: zwcai@hkbu.edu.hk

Contents

Experimental section..S3

Identification of putative lipids in dual-polarity modes..S4

Matrix deposition device..S33

Lipid profile obtained from tissues and cells..S34

Experimental section

Matrix coating

The matrix solution was sprayed through the capillary nozzle onto the ITO slide, and several parameters were carefully optimized to achieve the best results. These parameters include the electrospray voltage, the height of the spray tip above the sample surface, the solvent used, the flow rate of the matrix solution, and the temperature of the heating tube.

In addition, we observed that environmental conditions, particularly humidity, played a significant role in the deposition process. Under high humidity conditions, specifically when the relative humidity exceeded 80%, we found that increasing the nozzle temperature to 90°C expedited the drying process and prevented excessive crystal growth. Furthermore, the 30% water content in the solvent mixture contributed to the formation of smaller NEDC crystals, which further improved the uniformity of the matrix coating. These adjustments were critical for achieving consistent performance across varying environmental conditions and enhancing the reproducibility of the method.

Identification of putative lipids in dual-polarity modes

Table S1 Putative lipids detected by MALDI2-MSI from mouse kidney in positive ionization mode.

Exp. *m/z*	Cal. *m/z*	Tentatively identity	Delta (ppm)	Formula	Ion type
369.352	369.352	ST 27:1;O	0.27	$C_{27}H_{46}O$	$[M+H-H_2O]^+$
385.274	385.274	ST 25:4;O_4	0.26	$C_{25}H_{38}O_4$	$[M+H-H_2O]^+$
391.286	391.284	ST 24:1;O_5*	4.86	$C_{24}H_{40}O_5$	$[M+H-H_2O]^+$
419.257	419.256	LPA 18:1*	3.10	$C_{21}H_{41}O_7P$	$[M+H-H_2O]^+$
478.332	478.329	LPC O-16:2	4.81	$C_{24}H_{48}NO_6P$	$[M+H]^+$
479.335	479.337	ST 28:2;O_7	4.17	$C_{28}H_{48}O_7$	$[M+H-H_2O]]^+$
480.345	480.345	LPC O-16:1	0.21	$C_{24}H_{50}NO_6P$	$[M+H]^+$
482.325	482.324	LPE 18:0	2.70	$C_{23}H_{48}NO_7P$	$[M+H]^+$
482.363	482.361	LPC O-16:0	4.56	$C_{24}H_{52}NO_6P$	$[M+H]^+$
494.325	494.324	LPC 16:1	2.63	$C_{24}H_{48}NO_7P$	$[M+H]^+$
496.341	496.340	LPC 16:0	3.22	$C_{24}H_{50}NO_7P$	$[M+H]^+$
498.151	498.153	ST 22:6;O7;G	2.21	$C_{24}H_{29}NO_8K$	$[M+K]^+$
500.313	500.314	LPC O-18:5	1.00	$C_{26}H_{46}NO_6P$	$[M+H]^+$
500.486	500.483	Cer 34:3;O*	6.19	$C_{34}H_{63}NO_2$	$[M+H-H_2O]^+$
502.331	502.329	LPC O-18:4	3.38	$C_{26}H_{48}NO_6P$	$[M+H]^+$
503.334	503.334	ST 28:1;O_6	0.60	$C_{28}H_{48}O_6Na$	$[M+Na]^+$
504.347	504.345	LPC O-18:3	3.77	$C_{26}H_{50}NO_6P$	$[M+H]^+$
505.351	505.352	DG 27:5;O	-1.98	$C_{30}H_{48}O_6$	$[M+H]^+$
506.363	506.361	LPC O-18:2	5.13	$C_{26}H_{52}NO_6P$	$[M+H]^+$
508.377	508.376	LPC O-18:1	2.36	$C_{26}H_{54}NO_6P$	$[M+H]^+$
510.356	510.355	LPE 20:0	1.57	$C_{25}H_{52}NO_7P$	$[M+H]^+$
518.326	518.324	LPC 18:3	3.28	$C_{26}H_{48}NO_7P$	$[M+H]^+$

518.494	518.493	Cer 34:2;O_2	2.12	$C_{34}H_{65}NO_3$	$[M+H-H_2O]^+$
520.341	520.340	LPC 18:2	1.92	$C_{26}H_{50}NO_7P$	$[M+H]^+$
520.510	520.509	Cer 34:2;O	2.11	$C_{34}H_{65}NO_2$	$[M+H]^+$
522.356	522.355	LPC 18:1	0.77	$C_{26}H_{52}NO_7P$	$[M+H]^+$
522.526	522.524	Cer 34:1;O*	2.30	$C_{34}H_{67}NO_2$	$[M+H]^+$
524.373	524.371	LPC 18:0	4.20	$C_{26}H_{54}NO_7P$	$[M+H]^+$
526.330	526.329	CerP 28:5;O_2	2.09	$C_{28}H_{48}NO_6P$	$[M+H]^+$
528.345	528.345	LPC O-20:5	0.19	$C_{28}H_{50}NO_6P$	$[M+H]^+$
530.362	530.361	LPC O-20:4	1.89	$C_{28}H_{52}NO_6P$	$[M+H]^+$
534.295	534.296	LPE O-22:6	0.19	$C_{27}H_{46}NO_6PNa$	$[M+Na]^+$
538.388	538.387	LPE 22:0	1.86	$C_{27}H_{56}NO_7P$	$[M+H]^+$
540.478	540.478	Cer 36:6;O	0.93	$C_{36}H_{61}NO_2$	$[M+H]^+$
542.492	542.493	Cer 36:5;O	1.47	$C_{36}H_{63}NO_2$	$[M+H]^+$
544.340	544.340	LPC 20:4	0.18	$C_{28}H_{50}NO_7P$	$[M+H]^+$
546.355	546.355	LPC 20:3	0.73	$C_{28}H_{52}NO_7P$	$[M+H]^+$
550.330	550.329	LPC 22:6	2.18	$C_{30}H_{50}NO_7P$	$[M+H-H2O]^+$
551.504	551.503	DG O-32:2	0.91	$C_{35}H_{66}O_4$	$[M+H]^+$
559.176	559.176	ST 27:6;O_5;S	0.72	$C_{27}H_{36}O_8SK$	$[M+K]^+$
559.469	559.472	DG O-33:5	4.83	$C_{36}H_{62}O_4$	$[M+H]^+$
563.332	563.334	LPG 22:3	4.44	$C_{28}H_{51}O_9P$	$[M+H]^+$
563.540	563.540	DG O-34:1	0.89	$C_{37}H_{72}O_4$	$[M+H-H_2O]^+$
564.251	564.249	LPE 22:6	3.72	$C_{27}H_{44}NO_7PK$	$[M+K]^+$
573.489	573.488	CE 10:0;O_2	2.27	$C_{37}H_{64}O_4$	$[M+H]^+$
575.506	575.503	DG O-34:4	3.65	$C_{37}H_{66}O_4$	$[M+H]^+$
577.520	577.519	DG O-34:3	2.42	$C_{37}H_{68}O_4$	$[M+H]^+$
578.205	578.203	ST 24:5;O_8;T	3.80	$C_{26}H_{37}NO_{10}SNa$	$[M+Na]^+$
583.508	583.509	CE 12:1;O	0.17	$C_{39}H_{66}O_3$	$[M+H]^+$
585.525	585.524	CE 12:0;O	1.54	$C_{39}H_{68}O_3$	$[M+H]^+$
586.592	586.592	Cer 40:2;O	0.85	$C_{40}H_{77}NO_2$	$[M+H-H_2O]^+$

597.488	597.488	CE 12:2;O_2	0.17	$C_{39}H_{64}O_4$	$[M+H]^+$
598.555	598.556	Cer 40:5;O	0.84	$C_{40}H_{71}NO_2$	$[M+H]^+$
601.516	601.519	CE 12:0;O_2	4.32	$C_{39}H_{68}O_4$	$[M+H]^+$
603.535	603.535	DG O-36:4	0.17	$C_{39}H_{70}O_4$	$[M+H]^+$
607.510	607.509	CE 14:3;O	2.63	$C_{41}H_{66}O_3$	$[M+H]^+$
609.524	609.524	CE 14:2;O	0.33	$C_{41}H_{68}O_3$	$[M+H]^+$
611.539	611.540	CE 14:1;O	1.31	$C_{41}H_{70}O_3$	$[M+H]^+$
612.610	612.608	Cer 42:3;O	3.75	$C_{42}H_{79}NO_2$	$[M+H-H_2O]^+$
618.622	618.618	Cer 41:1;O_2	5.33	$C_{41}H_{81}NO_3$	$[M+H-H_2O]^+$
620.480	620.480	Cer 39:6;O	0.16	$C_{39}H_{67}NO_2K$	$[M+K]^+$
621.486	621.488	CE 14:4;O_2	2.41	$C_{41}H_{64}O_4$	$[M+H]^+$
623.503	623.503	CE 14:3;O_2	0.48	$C_{41}H_{66}O_4$	$[M+H]^+$
625.520	625.519	CE 14:2;O_2	2.08	$C_{41}H_{68}O_4$	$[M+H]^+$
626.585	626.587	Cer 42:5;O	3.83	$C_{42}H_{75}NO_2$	$[M+H]^+$
627.535	627.535	CE 14:1;O_2	0.32	$C_{41}H_{70}O_4$	$[M+H]^+$
628.602	628.603	Cer 42:4;O*	0.48	$C_{42}H_{77}NO_2$	$[M+H]^+$
632.635	632.634	Cer 42:2;O	1.74	$C_{42}H_{81}NO_2$	$[M+H]^+$
633.525	633.524	CE 16:4;O	0.63	$C_{43}H_{68}O_3$	$[M+H]^+$
635.539	635.540	CE 16:3;O	0.94	$C_{43}H_{70}O_3$	$[M+H]^+$
637.554	637.555	CE 16:2;O	2.51	$C_{43}H_{72}O_3$	$[M+H]^+$
640.602	640.603	Cer 43:5;O	0.78	$C_{43}H_{77}NO_2$	$[M+H]^+$
649.520	649.519	CE 16:4;O_2	1.54	$C_{43}H_{68}O_4$	$[M+H]^+$
651.537	651.535	CE 16:3;O_2	3.07	$C_{43}H_{70}O_4$	$[M+H]^+$
653.547	653.548	DG O-38:4	2.14	$C_{41}H_{74}O_4Na$	$[M+Na]^+$
653.605	653.608	DG 38:0*	3.82	$C_{41}H_{80}O_5$	$[M+H]^+$
655.471	655.470	LPA 34:4*	1.68	$C_{37}H_{67}O_7P$	$[M+H]^+$
655.567	655.566	CE 16:1;O_2	1.22	$C_{43}H_{74}O_4$	$[M+H]^+$
664.469	664.470	CerP 38:6;O_2	1.05	$C_{38}H_{66}NO_6P$	$[M+H]^+$
666.486	666.486	CerP 38:5;O_2	0.00	$C_{38}H_{68}NO_6P$	$[M+H]^+$

667.530	667.530	CE 16:3;O_3	0.00	$C_{43}H_{70}O_5$	$[M+H]^+$
673.517	673.517	DG O-40:8	1.04	$C_{43}H_{70}O_4Na$	$[M+Na]^+$
680.443	680.442	CerP 36:3;O_2	1.62	$C_{36}H_{68}NO_6PK$	$[M+K]^+$
682.459	682.457	CerP 36:2;O_2	3.81	$C_{36}H_{70}NO_6PK$	$[M+K]^+$
683.501	683.501	PA 36:2	0.00	$C_{39}H_{73}O_8P$	$[M+H]^+$
684.462	684.460	PE 32:4	3.51	$C_{37}H_{66}NO_8P$	$[M+H-H_2O]^+$
684.505	684.505	HexCer 32:3;O_3	1.17	$C_{38}H_{69}NO_9$	$[M+H]^+$
689.560	689.559	CerPE 36:1;O_2	1.02	$C_{38}H_{77}N_2O_6P$	$[M+H]^+$
697.480	697.480	PA 36:4	0.72	$C_{39}H_{69}O_8P$	$[M+H]^+$
699.481	699.484	CerPE 34:1;O_2	4.00	$C_{36}H_{73}N_2O_6PK$	$[M+K]^+$
701.558	701.559	SM 34:2;O_2	1.71	$C_{39}H_{77}N_2O_6P$	$[M+H]^+$
703.574	703.575	SM 34:1;O_2	0.57	$C_{39}H_{79}N_2O_6P$	$[M+H]^+$
705.590	705.591	SM 34:0;O_2*	1.42	$C_{39}H_{81}N_2O_6P$	$[M+H]^+$
707.507	707.510	CerPE 36:3;O_2	3.96	$C_{38}H_{73}N_2O_6PNa$	$[M+Na]^+$
709.516	709.513	CerPE 34:1;O_5	5.36	$C_{36}H_{73}N_2O_9P$	$[M+H]^+$
710.489	710.489	CerP 38:2;O_2*	0.84	$C_{38}H_{74}NO_6PK$	$[M+K]^+$
711.438	711.436	PA 34:2	2.81	$C_{37}H_{69}O_8PK$	$[M+K]^+$
711.495	711.492	SM 30:1;O_6	4.50	$C_{35}H_{71}N_2O_{10}P$	$[M+H]^+$
711.545	711.544	CerPE 38:4;O_2	1.97	$C_{40}H_{75}N_2O_6P$	$[M+H]^+$
713.454	713.452	PA 34:1	3.64	$C_{37}H_{71}O_8PK$	$[M+K]^+$
715.455	715.455	PG 32:4	0.56	$C_{38}H_{67}O_{10}P$	$[M+H]^+$
717.594	717.591	CerPE	4.46	$C_{40}H_{81}N_2O_6P$	$[M+H]^+$

		38:1;O_2			
720.556	720.554	PE 34:0	3.47	$C_{39}H_{78}NO_8P$	$[M+H]^+$
720.592	720.590	PC O-32:0	2.91	$C_{40}H_{82}NO_7P$	$[M+H]^+$
721.483	721.480	PA 38:6	3.47	$C_{41}H_{69}O_8P$	$[M+H]^+$
722.548	722.548	CerP 42:5;O_2	0.69	$C_{42}H_{76}NO_6P$	$[M+H]^+$
723.495	723.496	PA 38:5	1.24	$C_{41}H_{71}O_8P$	$[M+H]^+$
723.542	723.544	SM 36:5;O_2	2.07	$C_{41}H_{75}N_2O_6P$	$[M+H]^+$
724.498	724.499	HexCer 34:5;O_4	2.35	$C_{40}H_{69}NO_{10}$	$[M+H]^+$
724.530	724.528	PE O-36:5*	3.73	$C_{41}H_{74}NO_7P$	$[M+H]^+$
725.560	725.559	CerPE 39:4;O_2	0.96	$C_{41}H_{77}N_2O_6P$	$[M+H]^+$
727.467	727.467	PA O-38:7	0.55	$C_{41}H_{69}O_7PNa$	$[M+Na]^+$
728.519	728.523	PC 32:3	4.94	$C_{40}H_{74}NO_8P$	$[M+H]^+$
729.481	729.481	CerPE 36:5;O_5	0.14	$C_{38}H_{69}N_2O9P$	$[M+H]^+$
731.497	731.497	CerPE 36:4;O_5	0.41	$C_{38}H_{71}N_2O_9P$	$[M+H]^+$
731.607	731.606	SM 36:1;O_2*	1.37	$C_{41}H_{83}N_2O_6P$	$[M+H]^+$
732.555	732.554	PC 32:1	1.77	$C_{40}H_{78}NO_8P$	$[M+H]^+$
734.569	734.569	PC 32:0	0.95	$C_{40}H_{80}NO_8P$	$[M+H]^+$
735.436	735.432	CerPE 35:6;O_5	5.44	$C_{37}H_{65}N_2O_9PNa$	$[M+Na]^+$
736.577	736.572	HexCer 37:4;O_2	6.25	$C_{43}H_{77}NO_8$	$[M+H]^+$
737.454	737.452	PA 36:3	2.85	$C_{39}H_{71}O_8PK$	$[M+K]^+$
737.579	737.580	SM 34:0;O_4*	1.36	$C_{39}H_{81}N_2O_8P$	$[M+H]^+$
738.520	738.520	CerP 40:2;O_2	0.68	$C_{40}H_{78}NO_6PK$	$[M+K]^+$
739.466	739.468	PA 36:2	1.76	$C_{39}H_{73}O_8PK$	$[M+K]^+$

740.524	740.523	PE 36:4	2.57	$C_{41}H_{74}NO_8P$	$[M+H]^+$
741.480	741.481	SM 34:6;O_5	1.35	$C_{39}H_{69}N_2O_9P$	$[M+H]^+$
742.482	742.478	PC 30:1	4.71	$C_{38}H_{74}NO_8PK$	$[M+K]^+$
743.537	743.533	CerPE 38:4;O_4	4.84	$C_{40}H_{75}N_2O_8P$	$[M+H]^+$
744.553	744.554	PE 36:2	1.61	$C_{41}H_{78}NO_8P$	$[M+H]^+$
744.590	744.590	PC O-34:2	0.40	$C_{42}H_{82}NO_7P$	$[M+H]^+$
745.480	745.480	PA 40:8	0.13	$C_{43}H_{69}O_8P$	$[M+H]^+$
746.569	746.569	PE 36:1	0.00	$C_{41}H_{80}NO_8P$	$[M+H]^+$
746.607	746.606	PC O-34:1	1.21	$C_{42}H_{84}NO_7P$	$[M+H]^+$
748.588	748.585	PE 36:0	3.61	$C_{41}H_{82}NO_8P$	$[M+H]^+$
749.509	749.512	PA 40:6	3.20	$C_{43}H_{73}O_8P$	$[M+H]^+$
750.578	750.577	CerP 42:2;O_2	1.07	$C_{42}H_{82}NO_6PNa$	$[M+Na]^+$
751.524	751.525	PA 38:2	0.67	$C_{41}H_{77}O_8PNa$	$[M+Na]^+$
752.558	752.559	PE O-38:5*	0.80	$C_{43}H_{78}NO_7P$	$[M+H]^+$
753.485	753.485	PA 42:9*	0.53	$C_{45}H_{71}O_8P$	$[M+H-H_2O]^+$
753.590	753.591	SM 38:4;O_2	0.53	$C_{43}H_{81}N_2O_6P$	$[M+H]^+$
754.480	754.477	SHexCer 31:1;O3	3.71	$C_{37}H_{71}NO_{12}S$	$[M+H]^+$
754.540	754.538	PC 34:4	2.78	$C_{42}H_{76}NO_8P$	$[M+H]^+$
755.499	755.501	PA 42:8*	2.65	$C_{45}H_{73}O_8P$	$[M+H-H_2O]^+$
756.554	756.554	PC 34:3	0.13	$C_{42}H_{78}NO_8P$	$[M+H]^+$
757.421	757.421	PA 38:7	0.00	$C_{41}H_{67}O_8PK$	$[M+K]^+$
757.508	757.510	CerPE 36:2;O_5	3.43	$C_{38}H_{75}N_2O_9PNa$	$[M+Na]^+$
758.512	758.512	PC O-36:9	0.00	$C_{44}H_{72}NO_7P$	$[M+H]^+$
758.569	758.569	PC 34:2	0.79	$C_{42}H_{80}NO_8P$	$[M+H]^+$
759.442	759.444	PI 30:3*	3.03	$C_{39}H_{69}O_{13}P$	$[M+H-H_2O]^+$
759.639	759.637	SM 38:1;O_2*	2.37	$C_{43}H_{87}N_2O_6P$	$[M+H]^+$

760.586	760.585	PC 34:1	0.53	$C_{42}H_{82}NO_8P$	$[M+H]^+$
761.457	761.460	PI 30:2*	3.80	$C_{39}H_{71}O_{13}P$	$[M+H-H_2O]^+$
762.486	762.482	SHexCer 33:3;O_2	4.46	$C_{39}H_{71}NO_{11}S$	$[M+H]^+$
762.505	762.507	PE 38:7	2.89	$C_{43}H_{72}NO_8P$	$[M+H]^+$
762.600	762.601	PC 34:0	1.05	$C_{42}H_{84}NO_8P$	$[M+H]^+$
765.484	765.483	PA 38:3	1.44	$C_{41}H_{75}O_8PK$	$[M+K]^+$
766.541	766.538	PE 38:5	4.04	$C_{43}H_{76}NO_8P$	$[M+H]^+$
766.572	766.575	PC O-36:5	3.39	$C_{44}H_{80}NO_7P$	$[M+H]^+$
767.496	767.495	SM 34:4;O_5	2.08	$C_{39}H_{73}N_2O_9PNa$	$[M+Na]^+$
768.552	768.554	PE 38:4	2.08	$C_{43}H_{78}NO_8P$	$[M+H]^+$
768.587	768.590	PC O-36:4	4.42	$C_{44}H_{82}NO_7P$	$[M+H]^+$
769.479	769.480	PA 42:10	1.82	$C_{45}H_{69}O_8P$	$[M+H]^+$
769.560	769.559	TG 42:4;O_2	1.04	$C_{45}H_{78}O_8Na$	$[M+Na]^+$
770.569	770.569	PE 38:3	1.17	$C_{43}H_{80}NO_8P$	$[M+H]^+$
770.603	770.606	PC O-36:3	3.50	$C_{44}H_{84}NO_7P$	$[M+H]^+$
771.474	771.469	SM 33:3;O_5*	6.49	$C_{38}H_{73}N_2O_9PK$	$[M+K]^+$
771.494	771.496	PA 42:9	2.85	$C_{45}H_{71}O_8P$	$[M+H]^+$
772.528	772.528	PE O-40:9	0.78	$C_{45}H_{74}NO_7P$	$[M+H]^+$
772.586	772.585	PE 38:2	1.29	$C_{43}H_{82}NO_8P$	$[M+H]^+$
773.479	773.475	PG O-38:10	4.91	$C_{44}H_{69}O_9P$	$[M+H]^+$
774.543	774.543	PE O-40:8*	0.00	$C_{45}H_{76}NO_7P$	$[M+H]^+$
774.601	774.601	PE 38:1	0.77	$C_{43}H_{84}NO_8P$	$[M+H]^+$
776.463	776.463	PE 36:5	0.13	$C_{41}H_{72}NO_8PK$	[M+K]+
776.556	776.559	PE O-40:7	3.99	$C_{45}H_{78}NO_7P$	$[M+H]^+$
776.595	776.593	CerP 44:3;O_2	2.32	$C_{44}H_{84}NO_6PNa$	$[M+Na]^+$
778.480	778.477	SHexCer 33:3;O_3	3.98	$C_{39}H_{71}NO_{12}S$	$[M+H]^+$
778.539	778.538	PC 36:6	0.51	$C_{44}H_{76}NO_8P$	$[M+H]^+$

778.609	778.609	CerP 44:2;O_2	0.13	$C_{44}H_{86}NO_6PNa$	$[M+Na]^+$
780.491	780.494	PE 36:3	3.59	$C_{41}H_{76}NO_8PK$	$[M+K]^+$
780.554	780.554	PC 36:5	0.13	$C_{44}H_{78}NO_8P$	$[M+H]^+$
782.511	782.508	SHexCer 33:1;O_3	3.96	$C_{39}H_{75}NO_{12}S$	$[M+H]^+$
782.570	782.569	PC 36:4	0.89	$C_{44}H_{80}NO_8P$	$[M+H]^+$
784.525	784.524	SHexCer 33:0;O_3	1.40	$C_{39}H_{77}NO_{12}S$	$[M+H]^+$
784.584	784.585	PC 36:3	1.53	$C_{44}H_{82}NO_8P$	$[M+H]^+$
785.455	785.452	PA 40:7	4.33	$C_{43}H_{71}O_8PK$	$[M+K]^+$
785.459	785.460	PI 32:4*	1.15	$C_{41}H_{71}O_{13}P$	$[M+H-H_2O]^+$
786.603	786.601	PC 36:2	2.67	$C_{44}H_{84}NO_8P$	$[M+H]^+$
787.465	787.463	SM 33:3;O_6	1.52	$C_{38}H_{73}N_2O_{10}PK$	$[M+K]^+$
787.669	787.669	SM 40:1;O_2	0.89	$C_{45}H_{91}N_2O_6P$	$[M+H]^+$
788.521	788.523	PE 40:8	2.41	$C_{45}H_{74}NO_8P$	$[M+H]^+$
788.556	788.559	PC O-38:8	3.68	$C_{46}H_{78}NO_7P$	$[M+H]^+$
788.615	788.616	PC 36:1	1.52	$C_{44}H_{86}NO_8P$	$[M+H]^+$
789.483	789.483	PA 40:5	0.00	$C_{43}H_{75}O_8PK$	$[M+K]^+$
790.538	790.538	PE 40:7	0.38	$C_{45}H_{76}NO_8P$	$[M+H]^+$
792.592	792.590	PC O-38:6	2.14	$C_{46}H_{82}NO_7P$	$[M+H]^+$
794.512	794.508	SHexCer 34:2;O_3	5.03	$C_{40}H_{75}NO_{12}S$	$[M+H]^+$
794.604	794.606	PC O-38:5*	2.64	$C_{46}H_{84}NO_7P$	$[M+H]^+$
796.525	796.524	SHexCer 34:1;O_3	1.38	$C_{40}H_{77}NO_{12}S$	$[M+H]^+$
796.585	796.585	PE 40:4	0.50	$C_{45}H_{82}NO_8P$	$[M+H]^+$
798.543	798.540	SHexCer 34:0;O_3	3.88	$C_{40}H_{79}NO_{12}S$	$[M+H]^+$
800.462	800.461	SHexCer	0.25	$C_{41}H_{69}NO_{12}S$	$[M+H]^+$

		35:6;O_3			
800.554	800.552	HexCer 37:4;O_6	2.25	$C_{43}H_{77}NO_{12}$	$[M+H]^+$
800.618	800.616	PE 40:2	1.50	$C_{45}H_{86}NO_8P$	$[M+H]^+$
801.559	801.562	PG 36:0	3.37	$C_{42}H_{83}O_{10}PNa$	$[M+Na]^+$
801.683	801.684	SM 41:1;O_2*	1.50	$C_{46}H_{93}N_2O_6P$	$[M+H]^+$
802.477	802.477	SHexCer 35:5;O_3	0.25	$C_{41}H_{71}NO_{12}S$	$[M+H]^+$
802.537	802.538	PC 38:8	0.87	$C_{46}H_{76}NO_8P$	$[M+H]^+$
802.630	802.632	PE 40:1	2.87	$C_{45}H_{88}NO_8P$	$[M+H]^+$
803.481	803.484	PG 34:1;O	3.24	$C_{40}H_{77}O_{11}PK$	$[M+K]^+$
804.498	804.494	PE O-38:6;O	4.97	$C_{43}H_{76}NO_8PK$	$[M+K]^+$
804.550	804.554	PC 38:7	4.35	$C_{46}H_{78}NO_8P$	$[M+H]^+$
806.512	806.508	SHexCer 35:3;O_3	4.22	$C_{41}H_{75}NO_{12}S$	$[M+H]^+$
806.568	806.569	PC 38:6	1.49	$C_{46}H_{80}NO_8P$	$[M+H]^+$
808.583	808.585	PC 38:5	-2.47	$C_{46}H_{82}NO_8P$	$[M+H]^+$
809.651	809.653	SM 42:4;O_2	2.47	$C_{47}H_{89}N_2O_6P$	$[M+H]^+$
810.603	810.601	PC 38:4	2.84	$C_{46}H_{84}NO_8P$	$[M+H]^+$
811.670	811.669	SM 42:3;O_2*	1.23	$C_{47}H_{91}N_2O_6P$	$[M+H]^+$
812.498	812.498	SHexCer 37:6;O_2	0.37	$C_{43}H_{73}NO_{11}S$	$[M+H]^+$
812.522	812.523	PE 42:10	0.12	$C_{47}H_{74}NO_8P$	$[M+H]^+$
812.612	812.616	PC 38:3	5.66	$C_{46}H_{86}NO_8P$	$[M+H]^+$
813.490	813.491	PI 34:4*	1.48	$C_{43}H_{75}O_{13}P$	$[M+H-H_2O]^+$
813.685	813.684	SM 42:2;O_2	0.86	$C_{47}H_{93}N_2O_6P$	$[M+H]^+$
814.516	814.513	SHexCer 37:5;O_2	2.58	$C_{43}H_{75}NO_{11}S$	$[M+H]^+$
814.577	814.575	PC O-40:9	2.58	$C_{48}H_{80}NO_7P$	$[M+H]^+$

814.691	814.692	Cer 51:6;O_5*	1.60	$C_{51}H_{91}NO_6$	$[M+H]^+$
815.702	815.700	SM 42:1;O_2	1.84	$C_{47}H_{95}N_2O_6P$	$[M+H]^+$
816.534	816.530	CerP 45:6;O_3	4.29	$C_{45}H_{80}NO_7PK$	$[M+K]^+$
816.591	816.590	PC O-40:8	1.10	$C_{48}H_{82}NO_7P$	$[M+H]^+$
818.512	818.508	SHexCer 36:4;O_3	4.89	$C_{42}H_{75}NO_{12}S$	$[M+H]^+$
818.606	818.606	PC O-40:7	0.73	$C_{48}H_{84}NO_7P$	$[M+H]^+$
820.524	820.524	SHexCer 36:3;O_3	0.37	$C_{42}H_{77}NO_{12}S$	$[M+H]^+$
820.590	820.593	HexCer 41:6;O_4	4.14	$C_{47}H_{81}NO_{10}$	$[M+H]^+$
820.623	820.622	PC O-40:6	1.46	$C_{48}H_{86}NO_7P$	$[M+H]^+$
822.541	822.540	SHexCer 36:2;O_3	1.82	$C_{42}H_{79}NO_{12}S$	$[M+H]^+$
822.599	822.601	PE 42:5	2.43	$C_{47}H_{84}NO_8P$	$[M+H]^+$
823.607	823.609	SM 40:2;O_2	2.43	$C_{45}H_{89}N_2O_6PK$	$[M+K]^+$
823.669	823.669	SM 43:4;O_2	0.00	$C_{48}H_{91}N_2O_6P$	$[M+H]^+$
824.559	824.555	SHexCer 36:1;O_3	4.37	$C_{42}H_{81}NO_{12}S$	$[M+H]^+$
824.617	824.616	PE 42:4	0.12	$C_{47}H_{86}NO_8P$	$[M+H]^+$
826.569	826.571	SHexCer 36:0;O_3	2.30	$C_{42}H_{83}NO_{12}S$	$[M+H]^+$
827.487	827.486	TG 46:11;O_2	1.21	$C_{49}H_{72}O_8K$	$[M+K]^+$
828.493	828.493	SHexCer 37:6;O_3	0.48	$C_{43}H_{73}NO_{12}S$	$[M+H]^+$
828.551	828.554	PC 40:9	-3.38	$C_{48}H_{78}NO_8P$	$[M+H]^+$
830.550	830.545	SHexCer 38:4;O_2	6.62	$C_{44}H_{79}NO_{11}S$	$[M+H]^+$
830.571	830.569	PC 40:8	1.93	$C_{48}H_{80}NO_8P$	$[M+H]^+$

832.554	832.558	SHexCer 36:0;O_2	4.32	$C_{42}H_{83}NO_{11}SNa$	$[M+Na]^+$
832.583	832.585	PC 40:7	2.04	$C_{48}H_{82}NO_8P$	$[M+H]^+$
833.587	833.590	TG 47:7;O_2	3.48	$C_{50}H_{82}O_8Na$	$[M+Na]^+$
833.654	833.653	SM 44:6;O_2	1.08	$C_{49}H_{89}N_2O_6P$	$[M+H]^+$
834.542	834.540	SHexCer 37:3;O_3	3.24	$C_{43}H_{79}NO_{12}S$	$[M+H]^+$
834.601	834.601	PC 40:6	0.00	$C_{48}H_{84}NO_8P$	$[M+H]^+$
835.603	835.606	TG 47:6;O_2	3.59	$C_{50}H_{84}O_8Na$	$[M+Na]^+$
835.670	835.669	SM 44:5;O_2	1.20	$C_{49}H_{91}N_2O_6P$	$[M+H]^+$
836.610	836.616	PC 40:5	7.29	$C_{48}H_{86}NO_8P$	$[M+H]^+$
838.575	838.575	PC O-42:11	0.00	$C_{50}H_{80}NO_7P$	$[M+H]^+$
838.629	838.632	PC 40:4	3.58	$C_{48}H_{88}NO_8P$	$[M+H]^+$
839.640	839.640	TG 49:7;O_2	0.83	$C_{52}H_{86}O_8$	$[M+H]^+$
842.601	842.599	HexCer 40:4;O_6	2.49	$C_{46}H_{83}NO_{12}$	$[M+H]^+$
844.469	844.469	Hex2Cer 30:6;O_5	0.47	$C_{42}H_{69}NO_{16}$	$[M+H]^+$
844.524	844.524	SHexCer 38:5;O_3	0.36	$C_{44}H_{77}NO_{12}S$	$[M+H]^+$
846.469	846.467	SHexCer 36:6;O_5*	2.36	$C_{42}H_{71}NO_{14}S$	$[M+H]^+$
846.537	846.540	SHexCer 38:4;O_3	3.54	$C_{44}H_{79}NO_{12}S$	$[M+H]^+$
847.545	847.548	PG 42:8	4.60	$C_{48}H_{79}O_{10}P$	$[M+H]^+$
848.558	848.555	SHexCer 38:3;O_3	3.30	$C_{44}H_{81}NO_{12}S$	$[M+H]^+$
848.617	848.616	PE 44:6	1.18	$C_{49}H_{86}NO_8P$	$[M+H]^+$
849.624	849.622	PG 40:1;O	2.47	$C_{46}H_{89}O_{11}P$	$[M+H]^+$

850.499	850.498	SHexCer 36:4;O_5	1.18	$C_{42}H_{75}NO_{14}S$	$[M+H]^+$
850.567	850.571	SHexCer 38:2;O_3	4.35	$C_{44}H_{83}NO_{12}S$	$[M+H]^+$
850.631	850.632	PE 44:5	1.18	$C_{49}H_{88}NO_8P$	$[M+H]^+$
854.570	854.569	PC 42:10	0.70	$C_{50}H_{80}NO_8P$	$[M+H]^+$
855.660	855.659	SM 43:4;O_4	1.87	$C_{48}H_{91}N_2O_8P$	$[M+H]^+$
856.585	856.585	PC 42:9	0.23	$C_{50}H_{82}NO_8P$	$[M+H]^+$
861.671	861.669	SM 42:2;O_5	2.09	$C_{47}H_{93}N_2O_9P$	$[M+H]^+$
863.685	863.685	CerPE 45:1;O_5	0.46	$C_{47}H_{95}N_2O_9P$	$[M+H]^+$
866.458	866.458	PS 40:10;O	0.12	$C_{46}H_{70}NO_{11}PNa$	$[M+Na]^+$
868.466	868.467	Hex2Cer 30:5;O_5	0.69	$C_{42}H_{71}NO_{16}Na$	$[M+Na]^+$
872.557	872.558	Hex2Cer 30:0;O_6	0.46	$C_{42}H_{81}NO_{17}$	$[M+H]^+$
874.501	874.498	SHexCer 38:6;O_5	3.20	$C_{44}H_{75}NO_{14}S$	$[M+H]^+$
874.571	874.571	SHexCer 40:4;O_3	5.03	$C_{46}H_{83}NO_{12}S$	$[M+H]^+$
881.675	881.674	CerPE 48:5;O_4*	0.79	$C_{50}H_{93}N_2O_8P$	$[M+H]^+$
883.692	883.690	CerPE 48:4;O_4*	2.15	$C_{50}H_{95}N_2O_8P$	$[M+H]^+$
885.669	885.669	CerPE 47:4;O_5	0.00	$C_{49}H_{93}N_2O_9P$	$[M+H]^+$
889.699	889.700	SM 44:2;O_5*	1.46	$C_{49}H_{97}N_2O_9P$	$[M+H]^+$
891.659	891.656	CerPE 47:4;O_4	2.69	$C_{49}H_{93}N_2O_8PNa$	$[M+Na]^+$

895.689	895.690	SM 46:5;O_4	0.89	$C_{51}H_{95}N_2O_8P$	$[M+H]^+$
909.549	909.549	PI 40:7	0.33	$C_{49}H_{81}O_{13}P$	$[M+H]^+$
909.612	909.609	CerPE 46:4;O_5	3.08	$C_{48}H_{91}N_2O_9PK$	$[M+K]^+$
909.671	909.669	CerPE 49:6;O_5	2.42	$C_{51}H_{93}N_2O_9P$	$[M+H]^+$
913.701	913.700	CerPE 49:4;O_5*	0.55	$C_{51}H_{97}N_2O_9P$	$[M+H]^+$
920.690	920.686	SHexCer 44:1;O_2*	4.78	$C_{50}H_{97}NO_{11}S$	$[M+H]^+$
925.522	925.520	PI 38:4	2.27	$C_{47}H_{83}O_{13}PK$	$[M+K]^+$
927.533	927.536	PI 38:3	3.23	$C_{47}H_{85}O_{13}PK$	$[M+K]^+$
930.673	930.670	SHexCer 45:3;O_2*	3.65	$C_{51}H_{95}NO_{11}S$	$[M+H]^+$
935.633	935.637	TG 55:12;O_2	4.38	$C_{58}H_{88}O_8Na$	$[M+Na]^+$
947.506	947.505	PI O-40:8;O	1.48	$C_{49}H_{81}O_{13}PK$	$[M+K]^+$
951.646	951.647	TG 59:16	1.26	$C_{62}H_{88}O_6Na$	$[M+Na]^+$
952.655	952.654	SHexCer 47:6;O_2	1.26	$C_{53}H_{93}NO_{11}S$	$[M+H]^+$
953.666	953.663	TG 59:15	2.94	$C_{62}H_{90}O_6Na$	$[M+Na]^+$
954.672	954.672	Hex2Cer 37:0;O_5	0.00	$C_{49}H_{95}NO_{16}$	$[M+H]^+$
956.684	956.686	SHexCer 47:4;O_2*	1.46	$C_{53}H_{97}NO_{11}S$	$[M+H]^+$
966.671	966.670	SHexCer 48:6;O_2*	1.14	$C_{54}H_{95}NO_{11}S$	$[M+H]^+$
968.689	968.686	SHexCer 48:5;O_2*	3.30	$C_{54}H_{97}NO_{11}S$	$[M+H]^+$
970.701	970.701	SHexCer	0.41	$C_{54}H_{99}NO_{11}S$	$[M+H]^+$

		48:4;O_2*			
972.717	972.717	SHexCer 48:3;O_2*	0.31	$C_{54}H_{101}NO_{11}S$	$[M+H]^+$
981.742	981.740	CerPE 52:2;O_4*	2.04	$C_{54}H_{107}N_2O_8PK$	$[M+K]^+$
990.672	990.672	Hex2Cer 40:3;O_5	0.71	$C_{52}H_{95}NO_{16}$	$[M+H]^+$
991.678	991.679	TG 62:17	0.81	$C_{65}H_{92}O_6Na$	$[M+Na]^+$
992.690	992.688	Hex2Cer 40:2;O_5	1.81	$C_{52}H_{97}NO_{16}$	$[M+H]^+$
994.702	994.701	SHexCer 50:6;O_2*	0.80	$C_{56}H_{99}NO_{11}S$	$[M+H]^+$
995.707	995.710	TG 62:15	2.71	$C_{65}H_{96}O_6Na$	$[M+Na]^+$
996.718	996.719	Hex2Cer 40:0;O_5	1.00	$C_{52}H_{101}NO_{16}$	$[M+H]^+$
997.726	997.726	TG 62:14*	0.10	$C_{65}H_{98}O_6Na$	$[M+Na]^+$
998.664	998.660	SHexCer 48:6;O_4	4.11	$C_{54}H_{95}NO_{13}S$	$[M+H]^+$
1562.113	1562.105	CL 80:8*	5.06	$C_{89}H_{158}O_{17}P_2$	$[M+H]^+$
1568.148	1568.152	CL 80:5*	2.30	$C_{89}H_{164}O_{17}P_2$	$[M+H]^+$
1586.111	1586.105	CL 82:10*	3.66	$C_{91}H_{158}O_{17}P_2$	$[M+H]^+$
1588.122	1588.120	CL 82:9*	0.88	$C_{91}H_{160}O_{17}P_2$	$[M+H]^+$
1592.161	1592.152	CL 82:7*	5.59	$C_{91}H_{164}O_{17}P_2$	$[M+H]^+$
1594.176	1594.167	CL 82:6*	5.27	$C_{91}H_{166}O_{17}P_2$	$[M+H]^+$

*:not imaged in dual-polarity MALDI-MSI strategy with a single matrix application step

Table S2 Putative lipids detected by MALDI2-MSI from mouse kidney in negative ionization mode.

**Exp. *m/z***	**Cal. *m/z***	**Tentatively identity**	**Delta (ppm)**	**Formula**	**Ion type**
391.226	391.226	LPA O-16:2	0.00	$C_{19}H_{37}O_6P$	$[M-H]^-$
415.224	415.226	LPA O-18:4	2.65	$C_{21}H_{37}O_6P$	$[M-H]^-$
417.241	417.241	LPA O-18:3	0.24	$C_{21}H_{39}O_6P$	$[M-H]^-$
419.257	419.257	LPA O-18:2	0.48	$C_{21}H_{41}O_6P$	$[M-H]^-$
433.237	433.236	LPA 18:2	1.38	$C_{21}H_{39}O_7P$	$[M-H]^-$
436.284	436.283	LPE O-16:1	2.06	$C_{21}H_{44}NO_6P$	$[M-H]^-$
437.267	437.267	LPA 18:0	0.69	$C_{21}H_{43}O_7P$	$[M-H]^-$
452.279	452.278	LPE 16:0	1.33	$C_{21}H_{44}NO_7P$	$[M-H]^-$
457.236	457.236	LPA 20:4	0.87	$C_{23}H_{39}O_7P$	$[M-H]^-$
460.284	460.283	LPE O-18:3	1.74	$C_{23}H_{44}NO_6P$	$[M-H]^-$
462.300	462.299	LPE O-18:2	2.16	$C_{23}H_{46}NO_6P$	$[M-H]^-$
464.315	464.315	LPE O-18:1	1.51	$C_{23}H_{48}NO_6P$	$[M-H]^-$
466.330	466.330	LPE O-18:0	0.21	$C_{23}H_{50}NO_6P$	$[M-H]^-$
476.279	476.278	LPE 18:2	1.68	$C_{23}H_{44}NO_7P$	$[M-H]^-$
478.293	478.294	LPE 18:1	1.46	$C_{23}H_{46}NO_7P$	$[M-H]^-$
480.311	480.310	LPE 18:0	3.12	$C_{23}H_{48}NO_7P$	$[M-H]^-$
483.272	483.273	LPG 16:0	2.48	$C_{22}H_{45}O_9P$	$[M-H]^-$
489.262	489.262	LPG O-18:4	0.20	$C_{24}H_{43}O_8P$	$[M-H]^-$
500.278	500.278	LPE 20:4	0.60	$C_{25}H_{44}NO_7P$	$[M-H]^-$
508.342	508.341	LPE 20:0	1.77	$C_{25}H_{52}NO_7P$	$[M-H]^-$
524.279	524.278	LPE 22:6	1.34	$C_{27}H_{44}NO_7P$	$[M-H]^-$
525.281	525.283	LPG 18:1;O	4.00	$C_{24}H_{47}O_{10}P$	$[M-H]^-$
526.293	526.294	LPE 22:5	0.95	$C_{27}H_{46}NO_7P$	$[M-H]^-$
527.192	527.190	LPI 12:2;O	3.03	$C_{21}H_{37}O_{13}P$	$[M-H]^-$
527.297	527.299	LPG 18:0;O	3.98	$C_{24}H_{49}O_{10}P$	$[M-H]^-$
528.310	528.310	LPE 22:4	1.51	$C_{27}H_{48}NO_7P$	$[M-H]^-$
553.277	553.278	LPI O-16:2	2.35	$C_{25}H_{47}O_{11}P$	$[M-H]^-$
571.288	571.289	LPI 16:0	1.23	$C_{25}H_{49}O_{12}P$	$[M-H]^-$
581.310	581.310	LPI O-18:2	0.34	$C_{27}H_{51}O_{11}P$	$[M-H]^-$
583.325	583.325	LPI O-18:1	1.20	$C_{27}H_{53}O_{11}P$	$[M-H]^-$
597.306	597.305	LPI 18:1	1.67	$C_{27}H_{51}O_{12}P$	$[M-H]^-$
599.322	599.320	LPI 18:0	2.84	$C_{27}H_{53}O_{12}P$	$[M-H]^-$
616.473	616.471	CerP 34:1;O_2	2.11	$C_{34}H_{68}NO_6P$	$[M-H]^-$
630.488	630.487	LPE O-30:2	1.43	$C_{35}H_{70}NO_6P$	$[M-H]^-$

640.471	640.471	CerP 36:3;O_2	0.47	$C_{36}H_{68}NO_6P$	$[M-H]^-$
642.488	642.487	CerP 36:2;O_2	2.49	$C_{36}H_{70}NO_6P$	$[M-H]^-$
647.468	647.466	PA 32:0	2.93	$C_{35}H_{69}O_8P$	$[M-H]^-$
671.466	671.466	PA 34:2	0.00	$C_{37}H_{69}O_8P$	$[M-H]^-$
673.484	673.481	PA 34:1	3.27	$C_{37}H_{71}O_8P$	$[M-H]^-$
675.494	675.497	PA 34:0	5.03	$C_{37}H_{73}O_8P$	$[M-H]^-$
679.470	679.471	PA O-36:5	0.59	$C_{39}H_{69}O_7P$	$[M-H]^-$
685.531	685.529	CerPE 36:2;O_2	2.77	$C_{38}H_{75}N_2O_6P$	$[M-H]^-$
687.546	687.545	CerPE 36:1;O_2	1.75	$C_{38}H_{77}N_2O_6P$	$[M-H]^-$
689.553	689.549	PA O-36:0	5.66	$C_{39}H_{79}O_7P$	$[M-H]^-$
695.467	695.466	PA 36:4	1.87	$C_{39}H_{69}O_8P$	$[M-H]^-$
697.480	697.481	PA 36:3	2.29	$C_{39}H_{71}O_8P$	$[M-H]^-$
699.499	699.497	PA 36:2	2.14	$C_{39}H_{73}O_8P$	$[M-H]^-$
700.528	700.529	CerP 39:2;O_3	1.71	$C_{39}H_{76}NO_7P$	$[M-H]^-$
701.512	701.513	PA 36:1	0.43	$C_{39}H_{75}O_8P$	$[M-H]^-$
703.522	703.528	PA 36:0	9.52	$C_{39}H_{77}O_8P$	$[M-H]^-$
705.487	705.487	PA O-38:6	0.99	$C_{41}H_{71}O_7P$	$[M-H]^-$
707.501	707.502	PA O-38:5	1.98	$C_{41}H_{73}O_7P$	$[M-H]^-$
714.511	714.508	PE 34:2	4.48	$C_{39}H_{74}NO_8P$	$[M-H]^-$
715.577	715.576	CerPE 38:1;O_2	0.84	$C_{40}H_{81}N_2O_6P$	$[M-H]^-$
716.525	716.524	PE 34:1	2.37	$C_{39}H_{76}NO_8P$	$[M-H]^-$
718.542	718.539	PE 34:0	3.34	$C_{39}H_{78}NO_8P$	$[M-H]^-$
719.466	719.466	PA 38:6	0.28	$C_{41}H_{69}O_8P$	$[M-H]^-$
720.470	720.469	HexCer 34:6;O_4	0.97	$C_{40}H_{67}NO_{10}$	$[M-H]^-$
720.546	720.542	HexCer 36:4;O_2	4.86	$C_{42}H_{75}NO_8$	$[M-H]^-$
721.483	721.481	PA 38:5	1.94	$C_{41}H_{71}O_8P$	$[M-H]^-$
722.515	722.513	PE O-36:5	3.18	$C_{41}H_{74}NO_7P$	$[M-H]^-$
723.499	723.497	PA 38:4	2.21	$C_{41}H_{73}O_8P$	$[M-H]^-$
725.509	725.513	PA 38:3	4.96	$C_{41}H_{75}O_8P$	$[M-H]^-$
726.515	726.516	HexCer 34:3;O_4	2.20	$C_{40}H_{73}NO_{10}$	$[M-H]^-$
726.544	726.544	PE O-36:3	0.96	$C_{41}H_{78}NO_7P$	$[M-H]^-$
726.580	726.581	CerP 42:2;O_2	0.96	$C_{42}H_{82}NO_6P$	$[M-H]^-$
728.595	728.596	CerP 42:1;O_2	1.37	$C_{42}H_{84}NO_6P$	$[M-H]^-$
730.576	730.576	PE O-36:1	0.00	$C_{41}H_{82}NO_7P$	$[M-H]^-$
731.501	731.498	CerPE 36:3;O_5	3.96	$C_{38}H_{73}N_2O_9P$	$[M-H]^-$
736.492	736.492	PE 36:5	0.27	$C_{41}H_{72}NO_8P$	$[M-H]^-$
736.529	736.529	CerP 42:5;O_3	0.41	$C_{42}H_{76}NO_7P$	$[M-H]^-$

738.509	738.508	PE 36:4	1.76	$C_{41}H_{74}NO_8P$	$[M-H]^-$
740.521	740.524	PE 36:3	3.92	$C_{41}H_{76}NO_8P$	$[M-H]^-$
742.541	742.539	PE 36:2	2.83	$C_{41}H_{78}NO_8P$	$[M-H]^-$
743.608	743.607	CerPE 40:1;O_2	0.40	$C_{42}H_{85}N_2O_6P$	$[M-H]^-$
744.553	744.555	PE 36:1	3.09	$C_{41}H_{80}NO_8P$	$[M-H]^-$
745.484	745.481	PA 40:7	3.35	$C_{43}H_{71}O_8P$	$[M-H]^-$
746.513	746.513	PE O-38:7	0.54	$C_{43}H_{74}NO_7P$	$[M-H]^-$
746.569	746.571	PE 36:0	1.47	$C_{41}H_{82}NO_8P$	$[M-H]^-$
747.499	747.497	PA 40:6	3.21	$C_{43}H_{73}O_8P$	$[M-H]^-$
747.518	747.518	PG 34:1	0.13	$C_{40}H_{77}O_{10}P$	$[M-H]^-$
748.528	748.529	PE O-38:6	1.07	$C_{43}H_{76}NO_7P$	$[M-H]^-$
750.544	750.544	PE O-38:5	0.13	$C_{43}H_{78}NO_7P$	$[M-H]^-$
752.558	752.560	PE O-38:4	2.26	$C_{43}H_{80}NO_7P$	$[M-H]^-$
762.512	762.508	PE 38:6	5.38	$C_{43}H_{74}NO_8P$	$[M-H]^-$
764.524	764.524	PE 38:5	0.65	$C_{43}H_{76}NO_8P$	$[M-H]^-$
766.541	766.539	PE 38:4	2.61	$C_{43}H_{78}NO_8P$	$[M-H]^-$
768.552	768.555	PE 38:3	3.51	$C_{43}H_{80}NO_8P$	$[M-H]^-$
769.502	769.503	PG 36:4	0.65	$C_{42}H_{75}O_{10}P$	$[M-H]^-$
769.552	769.550	CerPE 40:4;O_4	2.47	$C_{42}H_{79}N_2O_8P$	$[M-H]^-$
770.515	770.513	PE O-40:9	1.95	$C_{45}H_{74}NO_7P$	$[M-H]^-$
770.569	770.571	PE 38:2	2.60	$C_{43}H_{82}NO_8P$	$[M-H]^-$
771.517	771.518	PG 36:3	1.81	$C_{42}H_{77}O_{10}P$	$[M-H]^-$
771.640	771.639	CerPE 42:1;O_2	2.20	$C_{44}H_{89}N_2O_6P$	$[M-H]^-$
772.528	772.529	PE O-40:8	0.65	$C_{45}H_{76}NO_7P$	$[M-H]^-$
772.586	772.586	PE 38:1	0.13	$C_{43}H_{84}NO_8P$	$[M-H]^-$
773.533	773.534	PG 36:2	0.90	$C_{42}H_{79}O_{10}P$	$[M-H]^-$
774.543	774.544	PE O-40:7	1.42	$C_{45}H_{78}NO_7P$	$[M-H]^-$
775.547	775.550	PG 36:1	3.35	$C_{42}H_{81}O_{10}P$	$[M-H]^-$
776.498	776.499	SHexCer 34:2;O_2	1.55	$C_{40}H_{75}NO_{11}S$	$[M-H]^-$
776.560	776.560	PE O-40:6	0.39	$C_{45}H_{80}NO_7P$	$[M-H]^-$
778.515	778.515	SHexCer 34:1;O_2	0.90	$C_{40}H_{77}NO_{11}S$	$[M-H]^-$
778.574	778.576	PE O-40:5	2.57	$C_{45}H_{82}NO_7P$	$[M-H]^-$
780.519	780.519	PE 38:5;O	0.00	$C_{43}H_{76}NO_9P$	$[M-H]^-$
780.558	780.555	CerP 44:5;O_4	3.46	$C_{44}H_{80}NO_8P$	$[M-H]^-$
782.500	782.498	PS 36:4	2.43	$C_{42}H_{74}NO_{10}P$	$[M-H]^-$
785.656	785.654	CerPE 43:1;O_2	1.78	$C_{45}H_{91}N_2O_6P$	$[M-H]^-$
786.528	786.529	PS 36:2	1.27	$C_{42}H_{78}NO_{10}P$	$[M-H]^-$

788.525	788.524	PE 40:7	1.14	$C_{45}H_{76}NO_8P$	$[M-H]^-$
788.544	788.545	PS 36:1	0.63	$C_{42}H_{80}NO_{10}P$	$[M-H]^-$
790.539	790.539	PE 40:6	0.89	$C_{45}H_{78}NO_8P$	$[M-H]^-$
791.542	791.544	PG 36:1;O	2.53	$C_{42}H_{81}O_{11}P$	$[M-H]^-$
792.552	792.555	PE 40:5	3.28	$C_{45}H_{80}NO_8P$	$[M-H]^-$
793.560	793.560	PG 36:0;O	0.63	$C_{42}H_{83}O_{11}P$	$[M-H]^-$
794.508	794.509	SHexCer 34:1;O_3	1.38	$C_{40}H_{77}NO_{12}S$	$[M-H]^-$
794.568	794.571	PE 40:4	3.27	$C_{45}H_{82}NO_8P$	$[M-H]^-$
796.633	796.631	HexCer 40:2;O_3	2.13	$C_{46}H_{87}NO_9$	$[M-H]^-$
797.653	797.654	CerPE 44:2;O_2	1.76	$C_{46}H_{91}N_2O_6P$	$[M-H]^-$
799.670	799.670	CerPE 44:1;O_2	0.50	$C_{46}H_{93}N_2O_6P$	$[M-H]^-$
800.498	800.499	SHexCer 36:4;O_2	1.62	$C_{42}H_{75}NO_{11}S$	$[M-H]^-$
800.558	800.560	PE O-42:8	3.00	$C_{47}H_{80}NO_7P$	$[M-H]^-$
802.481	802.478	SHexCer 35:4;O_3	3.86	$C_{41}H_{73}NO_{12}S$	$[M-H]^-$
802.578	802.576	PE O-42:7	2.37	$C_{47}H_{82}NO_7P$	$[M-H]^-$
804.591	804.591	PE O-42:6	0.99	$C_{47}H_{84}NO_7P$	$[M-H]^-$
806.497	806.498	PS 38:6	1.49	$C_{44}H_{74}NO_{10}P$	$[M-H]^-$
808.514	808.513	PS 38:5	1.24	$C_{44}H_{76}NO_{10}P$	$[M-H]^-$
809.518	809.519	PI 32:0	1.36	$C_{41}H_{79}O_{13}P$	$[M-H]^-$
810.530	810.529	PS 38:4	1.23	$C_{44}H_{78}NO_{10}P$	$[M-H]^-$
812.543	812.545	PS 38:3	2.46	$C_{44}H_{80}NO_{10}P$	$[M-H]^-$
816.656	816.657	HexCer 40:0;O_4	0.73	$C_{43}H_{79}NO_{11}S$	$[M-H]^-$
818.471	818.473	SHexCer 35:4;O_4*	1.95	$C_{41}H_{73}NO_{13}S$	$[M-H]^-$
818.570	818.571	PE 42:6	1.10	$C_{47}H_{82}NO_8P$	$[M-H]^-$
824.497	824.499	SHexCer 38:6;O_2	2.30	$C_{44}H_{75}NO_{11}S$	$[M-H]^-$
831.610	831.612	PG 40:1*	2.65	$C_{46}H_{89}O_{10}P$	$[M-H]^-$
832.513	832.513	PS 40:7	0.84	$C_{46}H_{76}NO_{10}P$	$[M-H]^-$
833.516	833.519	PI 34:2	2.64	$C_{43}H_{79}O_{13}P$	$[M-H]^-$
834.530	834.529	PS 40:6	0.84	$C_{46}H_{78}NO_{10}P$	$[M-H]^-$
834.576	834.577	SHexCer 38:1;O_2	1.68	$C_{44}H_{85}NO_{11}S$	$[M-H]^-$
836.543	836.545	PS 40:5	1.55	$C_{46}H_{80}NO_{10}P$	$[M-H]^-$
837.548	837.550	PI 34:0	2.39	$C_{43}H_{83}O_{13}P$	$[M-H]^-$
838.558	838.560	PS 40:4	3.22	$C_{46}H_{82}NO_{10}P$	$[M-H]^-$

840.531	840.530	SHexCer 39:5;O_2	0.59	$C_{45}H_{79}NO_{11}S$	$[M-H]^-$
843.537	843.539	PI O-36:4	3.08	$C_{45}H_{81}O_{12}P$	$[M-H]^-$
844.486	844.489	SHexCer 37:5;O_4	2.84	$C_{43}H_{75}NO_{13}S$	$[M-H]^-$
846.503	846.504	SHexCer 37:4;O_4	1.89	$C_{43}H_{77}NO_{13}S$	$[M-H]^-$
849.564	849.565	PG 42:6	1.18	$C_{48}H_{83}O_{10}P$	$[M-H]^-$
850.571	850.572	SHexCer 38:1;O_3	0.71	$C_{44}H_{85}NO_{12}S$	$[M-H]^-$
852.500	852.496	Hex2Cer 29:2;O_6	4.69	$C_{41}H_{75}NO_{17}$	$[M-H]^-$
852.577	852.576	CerP 47:6;O_6	1.06	$C_{47}H_{84}NO_{10}P$	$[M-H]^-$
857.519	857.519	PI 36:4	0.47	$C_{45}H_{79}O_{13}P$	$[M-H]^-$
859.532	859.534	PI 36:3	2.21	$C_{45}H_{81}O_{13}P$	$[M-H]^-$
860.590	860.593	SHexCer 40:2;O_2	2.90	$C_{46}H_{87}NO_{11}S$	$[M-H]^-$
861.550	861.550	PI 36:2	0.35	$C_{45}H_{83}O_{13}P$	$[M-H]^-$
862.606	862.608	SHexCer 40:1;O_2	2.43	$C_{46}H_{89}NO_{11}S$	$[M-H]^-$
863.564	863.566	PI 36:1	1.39	$C_{45}H_{85}O_{13}P$	$[M-H]^-$
864.588	864.588	SHexCer 39:1;O_3	0.69	$C_{45}H_{87}NO_{12}S$	$[M-H]^-$
869.557	869.555	PI O-38:5	1.96	$C_{47}H_{83}O_{12}P$	$[M-H]^-$
874.606	874.608	SHexCer 41:2;O_2	2.86	$C_{47}H_{89}NO_{11}S$	$[M-H]^-$
876.589	876.588	SHexCer 40:2;O_3	1.71	$C_{46}H_{87}NO_{12}S$	$[M-H]^-$
876.624	876.624	SHexCer 41:1;O_2	0.23	$C_{47}H_{91}NO_{11}S$	$[M-H]^-$
878.603	878.603	SHexCer 40:1;O_3	0.11	$C_{46}H_{89}NO_{12}S$	$[M-H]^-$
880.609	880.607	CerP 49:6;O_6	1.59	$C_{49}H_{88}NO_{10}P$	$[M-H]^-$
881.521	881.519	PI 38:6	2.27	$C_{47}H_{79}O_{13}P$	$[M-H]^-$
882.522	882.525	SHexCer 37:2;O_6	4.19	$C_{43}H_{81}NO_{15}S$	$[M-H]^-$
883.533	883.534	PI 38:5	1.70	$C_{47}H_{81}O_{13}P$	$[M-H]^-$
885.550	885.550	PI 38:4	0.45	$C_{47}H_{83}O_{13}P$	$[M-H]^-$
886.608	886.608	SHexCer 42:3;O_2	0.00	$C_{48}H_{89}NO_{11}S$	$[M-H]^-$
887.562	887.566	PI 38:3	3.94	$C_{47}H_{85}O_{13}P$	$[M-H]^-$
888.570	888.569	Hex2Cer	1.01	$C_{46}H_{83}NO_{15}$	$[M-H]^-$

		34:3;O_4			
888.623	888.624	SHexCer 42:2;O_2	0.90	$C_{48}H_{91}NO_{11}S$	$[M-H]^-$
890.580	890.585	Hex2Cer 34:2;O_4	4.83	$C_{46}H_{85}NO_{15}$	$[M-H]^-$
890.603	890.603	SHexCer 41:2;O_3	0.79	$C_{47}H_{89}NO_{12}S$	$[M-H]^-$
890.638	890.640	SHexCer 42:1;O_2	1.57	$C_{48}H_{93}NO_{11}S$	$[M-H]^-$
892.618	892.619	SHexCer 41:1;O_3	1.34	$C_{47}H_{91}NO_{12}S$	$[M-H]^-$
894.624	894.623	PS 44:4	0.89	$C_{50}H_{90}NO_{10}P$	$[M-H]^-$
902.604	902.603	SHexCer 42:3;O_3	0.22	$C_{48}H_{89}NO_{12}S$	$[M-H]^-$
904.619	904.619	SHexCer 42:2;O_3	0.44	$C_{48}H_{91}NO_{12}S$	$[M-H]^-$
905.623	905.628	PG 46:6	4.97	$C_{52}H_{91}O_{10}P$	$[M-H]^-$
906.634	906.635	SHexCer 42:1;O_3	1.21	$C_{48}H_{93}NO_{12}S$	$[M-H]^-$
908.639	908.639	CerP 51:6;O_6	0.88	$C_{51}H_{92}NO_{10}P$	$[M-H]^-$
909.549	909.550	PI 40:6	1.54	$C_{49}H_{83}O_{13}P$	$[M-H]^-$
911.561	911.566	PI 40:5	5.16	$C_{49}H_{85}O_{13}P$	$[M-H]^-$
913.582	913.581	PI 40:4	1.09	$C_{49}H_{87}O_{13}P$	$[M-H]^-$
915.594	915.597	PI 40:3	2.73	$C_{49}H_{89}O_{13}P$	$[M-H]^-$
918.671	918.671	SHexCer 44:1;O_2	0.22	$C_{50}H_{97}NO_{11}S$	$[M-H]^-$
920.649	920.647	HexCer 46:6;O_6	1.74	$C_{52}H_{91}NO_{12}$	$[M-H]^-$
920.676	920.675	CerP 53:6;O_5*	1.19	$C_{53}H_{96}NO_9P$	$[M-H]^-$
924.644	924.645	SHexCer 42:0;O_4	1.73	$C_{48}H_{95}NO_{13}S$	$[M-H]^-$
948.625	948.627	Hex2Cer 37:2;O_5	1.90	$C_{49}H_{91}NO_{16}$	$[M-H]^-$
950.638	950.640	SHexCer 47:6;O_2	1.89	$C_{53}H_{93}NO_{11}S$	$[M-H]^-$
952.655	952.655	SHexCer 47:5;O_2	0.10	$C_{53}H_{95}NO_{11}S$	$[M-H]^-$
965.517	965.516	PIP 38:4	0.41	$C_{47}H_{84}O_{16}P_2$	$[M-H]^-$
1013.422	1013.420	PIP2 36:6	2.27	$C_{45}H_{77}O_{19}P_3$	$[M-H]^-$
1042.673	1042.668	Hex2Cer 43:5;O_6	4.70	$C_{55}H_{97}NO_{17}$	$[M-H]^-$
1080.726	1080.720	Hex2Cer 47:6;O_5*	5.00	$C_{59}H_{103}NO_{16}$	$[M-H]^-$

1421.948	1421.949	CL 70:7	1.13	$C_{79}H_{140}O_{17}P_2$	$[M-H]^-$
1423.961	1423.965	CL 70:6	2.67	$C_{79}H_{142}O_{17}P_2$	$[M-H]^-$
1425.978	1425.981	CL 70:5	2.17	$C_{79}H_{144}O_{17}P_2$	$[M-H]^-$
1427.997	1427.996	CL 70:4	0.28	$C_{79}H_{146}O_{17}P_2$	$[M-H]^-$
1445.951	1445.949	CL 72:9	1.45	$C_{81}H_{140}O_{17}P_2$	$[M-H]^-$
1447.963	1447.965	CL 72:8	1.59	$C_{81}H_{142}O_{17}P_2$	$[M-H]^-$
1449.977	1449.981	CL 72:7	2.62	$C_{81}H_{144}O_{17}P_2$	$[M-H]^-$
1451.994	1451.996	CL 72:6	1.86	$C_{81}H_{146}O_{17}P_2$	$[M-H]^-$
1454.006	1454.012	CL 72:5	4.06	$C_{81}H_{148}O_{17}P_2$	$[M-H]^-$
1469.949	1469.949	CL 74:11	0.41	$C_{83}H_{140}O_{17}P_2$	$[M-H]^-$
1471.964	1471.965	CL 74:10	0.75	$C_{83}H_{142}O_{17}P_2$	$[M-H]^-$
1473.982	1473.981	CL 74:9	0.88	$C_{83}H_{144}O_{17}P_2$	$[M-H]^-$
1475.995	1475.996	CL 74:8	0.68	$C_{83}H_{146}O_{17}P_2$	$[M-H]^-$
1478.011	1478.012	CL 74:7	0.34	$C_{83}H_{148}O_{17}P_2$	$[M-H]^-$
1480.023	1480.028	CL 74:6	3.24	$C_{83}H_{150}O_{17}P_2$	$[M-H]^-$
1495.967	1495.965	CL 76:12	1.00	$C_{85}H_{142}O_{17}P_2$	$[M-H]^-$
1497.980	1497.981	CL 76:11	0.47	$C_{85}H_{144}O_{17}P_2$	$[M-H]^-$
1499.989	1499.996	CL 76:10	5.13	$C_{85}H_{146}O_{17}P_2$	$[M-H]^-$
1537.861	1537.856	PIP3 68:8	3.38	$C_{77}H_{138}O_{22}P_4$	$[M-H]^-$
1539.869	1539.871	PIP3 68:7	1.43	$C_{77}H_{140}O_{22}P_4$	$[M-H]^-$

*:not imaged in dual-polarity MALDI-MSI strategy with a single matrix application step

Table S3 Putative lipids detected by MALDI2-MSI from A549 cells in positive ionization mode.

**Exp. *m/z***	**Cal. *m/z***	**Tentatively identity**	**Delta (ppm)**	**Formula**	**Ion type**
496.339	496.340	LPC 16:0*	2.01	$C_{24}H_{50}NO_7P$	$[M+H]^+$
501.259	501.261	LPA 22:5;O	3.59	$C_{25}H_{41}O_8P$	$[M+H]^+$
503.266	503.262	LPI O-12:0	3.58	$C_{21}H_{43}O_{11}P$	$[M+H]^+$
504.344	504.345	LPC O-18:3	1.39	$C_{26}H_{50}NO_6P$	$[M+H]^+$
506.285	506.288	LPC 16:3;O	6.32	$C_{24}H_{44}NO_8P$	$[M+H]^+$
508.301	508.303	LPC 16:2;O	4.72	$C_{24}H_{46}NO_8P$	$[M+H]^+$
515.241	515.240	PA 22:5;O	0.19	$C_{25}H_{39}O_9P$	$[M+H]^+$
517.254	517.256	PA 22:4;O	4.45	$C_{25}H_{41}O_9P$	$[M+H]^+$
522.356	522.355	LPC 18:1*	0.57	$C_{26}H_{52}NO_7P$	$[M+H]^+$
535.167	535.171	LPI O-12:3	6.73	$C_{21}H_{37}O_{11}PK$	$[M+K]^+$
549.489	549.488	DG O-32:3	2.18	$C_{35}H_{64}O_4$	$[M+H]^+$
551.504	551.503	DG O-32:2	0.54	$C_{35}H_{66}O_4$	$[M+H]^+$
575.505	575.503	DG O-34:4	2.26	$C_{37}H_{66}O_4$	$[M+H]^+$
577.519	577.519	DG O-34:3	0.52	$C_{37}H_{68}O_4$	$[M+H]^+$
579.534	579.535	DG O-34:2	1.90	$C_{37}H_{70}O_4$	$[M+H]^+$
599.505	599.503	DG O-36:6	3.00	$C_{39}H_{66}O_4$	$[M+H]^+$
601.522	601.519	DG O-36:5	5.15	$C_{39}H_{68}O_4$	$[M+H]^+$
603.535	603.535	DG O-36:4	0.83	$C_{39}H_{70}O_4$	$[M+H]^+$
605.552	605.550	DG O-36:3	2.64	$C_{39}H_{72}O_4$	$[M+H]^+$
625.519	625.519	DG O-38:7*	0.80	$C_{41}H_{68}O_4$	$[M+H]^+$
627.532	627.535	DG O-38:6*	4.78	$C_{41}H_{70}O_4$	$[M+H]^+$
632.388	632.392	LPS 26:3	1.27	$C_{32}H_{58}NO_9P$	$[M+H]^+$
634.403	634.408	LPS 26:2	3.47	$C_{32}H_{60}NO_9P$	$[M+H]^+$
636.420	636.424	LPS 26:1	5.66	$C_{32}H_{62}NO_9P$	$[M+H]^+$

652.417	652.418	LPS 26:1;O	2.61	$C_{32}H_{62}NO_{10}P$	$[M+H]^+$
665.400	665.402	LPI O-24:3	3.46	$C_{33}H_{61}O_{11}P$	$[M+H]^+$
681.396	681.397	LPI 24:2	2.20	$C_{33}H_{61}O_{12}P$	$[M+H]^+$
683.407	683.413	LPI 24:1	0.88	$C_{33}H_{63}O_{12}P$	$[M+H]^+$
685.359	685.356	PI 22:1;O	5.11	$C_{31}H_{57}O_{14}P$	$[M+H]^+$
689.401	689.402	LPI O-26:5	1.74	$C_{35}H_{61}O_{11}P$	$[M+H]^+$
691.414	691.416	LPI O-26:4	4.32	$C_{35}H_{63}O_{11}P$	$[M+H]^+$
693.429	693.436	LPI O-26:3	7.21	$C_{35}H_{65}O_{11}P$	$[M+H]^+$
695.460	695.465	PA 36:5*	1.73	$C_{39}H_{67}O_8P$	$[M+H]^+$
697.476	697.478	PA 36:4	2.86	$C_{39}H_{69}O_8P$	$[M+H]^+$
707.410	707.405	PA 34:4	7.35	$C_{37}H_{65}O_8PK$	$[M+K]^+$
709.425	709.421	PA 34:3	6.06	$C_{37}H_{67}O_8PK$	$[M+K]^+$
711.441	711.436	PA 34:2	6.18	$C_{37}H_{69}O_8PK$	$[M+K]^+$
717.430	717.426	PA O-36:6	5.58	$C_{39}H_{67}O_7PK$	$[M+K]^+$
719.447	719.441	PA O-36:5	8.62	$C_{39}H_{69}O_7PK$	$[M+K]^+$
721.476	721.480	PA 38:6	6.65	$C_{41}H_{69}O_8P$	$[M+H]^+$
723.490	723.492	PA 38:5	2.76	$C_{41}H_{71}O_8P$	$[M+H]^+$
728.519	728.523	PC 32:3*	5.08	$C_{40}H_{74}NO_8P$	$[M+H]^+$
732.555	732.554	PC 32:1	1.77	$C_{40}H_{78}NO_8P$	$[M+H]^+$
733.424	733.429	PI 28:2*	6.95	$C_{37}H_{67}O_{13}P$	$[M+H-H_2O]^+$
734.571	734.569	PC 32:0	2.45	$C_{40}H_{80}NO_8P$	$[M+H]^+$
735.442	735.444	PI 28:1*	3.67	$C_{37}H_{69}O_{13}P$	$[M+H-H_2O]^+$
737.454	737.452	PA 36:3	2.58	$C_{39}H_{71}O_8PK$	$[M+K]^+$
739.470	739.468	PA 36:2	2.98	$C_{39}H_{73}O_8PK$	$[M+K]^+$
744.515	744.517	PC 32:3;O	2.82	$C_{40}H_{74}NO_9P$	$[M+H]^+$
746.566	746.569	PE 36:1	5.22	$C_{41}H_{80}NO_8P$	$[M+H]^+$
747.529	747.528	SM 34:3;O_5	1.20	$C_{39}H_{75}N_2O_9P$	$[M+H]^+$
749.511	749.512	PA 40:6	1.20	$C_{43}H_{73}O_8P$	$[M+H]^+$
754.534	754.538	PC 34:4*	5.96	$C_{42}H_{76}NO_8P$	$[M+H]^+$

756.551	756.554	PC 34:3*	3.57	$C_{42}H_{78}NO_8P$	$[M+H]^+$
758.398	758.401	PS 30:2;O	3.30	$C_{36}H_{66}NO_{11}PK$	$[M+K]^+$
758.570	758.569	PC 34:2	0.53	$C_{42}H_{80}NO_8P$	$[M+H]^+$
759.442	759.444	PI 30:3*	3.03	$C_{39}H_{69}O_{13}P$	$[M+H-H_2O]^+$
760.588	760.585	PC 34:1	3.16	$C_{42}H_{82}NO_8P$	$[M+H]^+$
761.359	761.361	PC 30:0 - $N(CH3)_3$	2.86	$C_{38}H_{76}NO_8PIn$	$[M+In]^+$
761.456	761.460	PI 30:2*	4.73	$C_{39}H_{71}O_{13}P$	$[M+H-H_2O]^+$
762.524	762.528	PS 34:1	5.90	$C_{40}H_{76}NO_{10}P$	$[M+H]^+$
762.596	762.601	PC 34:0*	5.90	$C_{42}H_{84}NO_8P$	$[M+H]^+$
764.539	764.544	PS 34:0	5.76	$C_{40}H_{78}NO_{10}P$	$[M+H]^+$
768.552	768.554	PE 38:4	2.21	$C_{43}H_{78}NO_8P$	$[M+H]^+$
775.381	775.381	PI 32:9*	0.90	$C_{39}H_{61}O_{12}PNa$	$[M+H-H_2O]^+$
780.550	780.554	PC 36:5	5.38	$C_{44}H_{78}NO_8P$	$[M+H]^+$
782.568	782.569	PC 36:4	2.30	$C_{44}H_{80}NO_8P$	$[M+H]^+$
784.580	784.585	PC 36:3	6.76	$C_{44}H_{82}NO_8P$	$[M+H]^+$
785.365	785.361	PC 32:2-$N(CH3)_3$	4.48	$C_{37}H_{67}O_8PIn$	$[M+In]^+$
786.604	786.601	PC 36:2	4.32	$C_{44}H_{84}NO_8P$	$[M+H]^+$
787.376	787.377	PC 32:1-$N(CH3)_3$	1.82	$C_{37}H_{69}O_8PIn$	$[M+In]^+$
789.392	789.393	PC 32:0-$N(CH3)_3$	1.11	$C_{37}H_{71}O_8PIn$	$[M+In]^+$
790.538	790.538	PE 40:7*	0.51	$C_{45}H_{76}NO_8P$	$[M+H]^+$
791.404	791.408	PA 34:0	5.60	$C_{37}H_{73}O_8P$	$[M+In]^+$
803.406	803.413	PI 34:9*	2.49	$C_{43}H_{65}O_{13}P$	$[M+H-H_2O]^+$
803.454	803.449	PG 38:10;O	5.97	$C_{44}H_{67}O_{11}P$	$[M+H]^+$
808.583	808.585	PC 38:5*	2.60	$C_{46}H_{82}NO_8P$	$[M+H]^+$
811.374	811.377	PC	3.86	$C_{39}H_{69}O_8PIn$	$[M+In]^+$

		34:3-N(CH3)$_3$			
813.390	813.393	PC 34:2-N(CH3)$_3$	2.68	$C_{39}H_{71}O_8PIn$	$[M+In]^+$
814.467	814.462	SHexCer 32:2;O$_6$*	5.89	$C_{38}H_{71}NO_{15}S$	[M+H]+
815.407	815.408	PC 34:1-N(CH3)3	1.63	$C_{39}H_{73}O_8PIn$	$[M+In]^+$
817.420	817.424	PC 34:0-N(CH3)$_3$	4.25	$C_{39}H_{75}O_8PIn$	$[M+In]^+$
820.434	820.435	PC 30:0	1.10	$C_{35}H_{67}O_8PIn$	$[M+In]^+$
830.421	830.419	PE 34:2	2.75	$C_{39}H_{74}NO_8PIn$	$[M+In]^+$
832.432	832.435	PE 34:1	3.21	$C_{39}H_{76}NO_8PIn$	$[M+In]^+$
834.452	834.450	PE 34:0	1.89	$C_{39}H_{78}NO_8PIn$	$[M+In]^+$
837.393	837.393	PC 36:4-N(CH3)$_3$	0.62	$C_{41}H_{71}O_8PIn$	$[M+In]^+$
838.423	838.420	Hex2Cer 28:6;O$_5$*	4.53	$C_{40}H_{65}NO_{16}Na$	$[M+Na]^+$
839.409	839.408	PC 36:3-N(CH3)$_3$	0.56	$C_{41}H_{73}O_8PIn$	$[M+In]^+$
840.438	840.435	Hex2Cer 28:5;O$_5$*	3.45	$C_{40}H_{67}NO_{16}Na$	$[M+Na]^+$
841.423	841.424	PC 36:2-N(CH3)$_3$	0.80	$C_{41}H_{75}O_8PIn$	$[M+In]^+$
842.491	842.493	SHexCer 34:2;O$_6$	2.14	$C_{40}H_{75}NO_{15}S$	$[M+H]^+$
843.437	843.440	PC 36:1-N(CH3)$_3$	2.88	$C_{41}H_{77}O_8PIn$	$[M+In]^+$
845.441	845.437	PG 38:8;O	5.09	$C_{44}H_{71}O_{11}PK$	$[M+K]^+$
846.450	846.450	PC 32:1	0.38	$C_{40}H_{78}NO_8PIn$	$[M+In]^+$
848.465	848.466	PC 32:0	1.85	$C_{40}H_{80}NO_8PIn$	$[M+In]^+$

856.435	856.430	Hex2Cer 28:5;O_6*	5.49	$C_{40}H_{67}NO_{17}Na$	$[M+Na]^+$
858.450	858.446	Hex2Cer 28:4;O_6	4.66	$C_{40}H_{69}NO_{17}Na$	$[M+Na]^+$
860.464	860.464	Hex2Cer 30:6;O_6	0.00	$C_{42}H_{69}NO_{17}$	$[M+H]^+$
862.475	862.480	Hex2Cer 30:5;O_6	5.22	$C_{42}H_{71}NO_{17}$	$[M+H]^+$
863.430	863.434	PI 36:10;O	4.40	$C_{45}H_{67}O_{14}P$	$[M+H]^+$
865.423	865.426	PI 34:6	3.93	$C_{43}H_{71}O_{13}PK$	$[M+K]^+$
867.434	867.442	PI 34:5	8.88	$C_{43}H_{73}O_{13}PK$	$[M+K]^+$
868.513	868.509	SHexCer 36:3;O_6	5.41	$C_{42}H_{77}NO_{15}S$	$[M+H]^+$
870.453	870.521	Hex2Cer 33:6;O_4	1.49	$C_{45}H_{75}NO_{15}$	$[M+H]^+$
872.470	872.466	PC 34:2	4.50	$C_{42}H_{80}NO_8PIn$	$[M+In]^+$
874.480	874.482	PC 34:1	2.42	$C_{42}H_{82}NO_8PIn$	$[M+In]^+$
876.422	876.495	Hex2Cer 31:5;O_6	1.94	$C_{43}H_{73}NO_{17}$	$[M+H]^+$
876.493	886.477	Hex2Cer 30:4;O_6	4.51	$C_{42}H_{73}NO_{17}Na$	$[M+Na]^+$
880.437	880.435	PE 38:5	2.42	$C_{43}H_{76}NO_8PIn$	$[M+In]^+$
882.451	882.450	PE 38:4	0.54	$C_{43}H_{78}NO_8PIn$	$[M+In]^+$
884.461	884.466	PE 38:3	5.96	$C_{43}H_{80}NO_8PIn$	$[M+In]^+$
886.481	886.482	PE 38:2	0.70	$C_{43}H_{82}NO_8PIn$	$[M+In]^+$
888.494	888.497	PE 38:1	3.91	$C_{43}H_{84}NO_8PIn$	$[M+In]^+$
894.454	894.450	PC 36:5	3.67	$C_{44}H_{78}NO_8PIn$	$[M+In]^+$
896.464	896.466	PC 36:4	2.31	$C_{44}H_{80}NO_8P$	$[M+In]^+$
898.479	898.482	PC 36:3	3.14	$C_{44}H_{82}NO_8PIn$	$[M+In]^+$
900.498	900.497	PC 36:2	0.92	$C_{44}H_{84}NO_8PIn$	$[M+In]^+$
902.441	902.440	PS 36:2	0.71	$C_{42}H_{78}NO_{10}PIn$	$[M+In]^+$
902.513	902.513	PC 36:1	0.09	$C_{44}H_{86}NO_8PIn$	$[M+In]^+$
904.451	904.456	PS 36:1	5.43	$C_{42}H_{80}NO_{10}PIn$	$[M+In]^+$
906.457	906.450	PE 40:6	6.82	$C_{45}H_{78}NO_8PIn$	$[M+In]^+$

926.439	926.440	PS 38:4	1.36	$C_{44}H_{78}NO_{10}PIn$	$[M+In]^+$
926.592	926.587	SHexCer 40:2;O_6	5.29	$C_{46}H_{87}NO_{15}S$	$[M+H]^+$
929.306	929.304	PC 34:1-N(CH3)$_3$	1.97	$C_{39}H_{72}O_8PIn_2$	$[M+2In-H]^+$
931.320	931.320	PC 34:0-N(CH3)$_3$	0.30	$C_{39}H_{74}O_8PIn_2$	$[M+2In-H]^+$
942.587	942.592	Hex2Cer 36:2;O_3	4.88	$C_{48}H_{89}NO_{14}K$	$[M+K]^+$
946.338	946.331	PE 34:1	7.07	$C_{39}H_{75}NO_8PIn_2$	$[M+2In-H]^+$
972.347	972.346	PE 36:2	0.96	$C_{41}H_{77}NO_8PIn_2$	$[M+2In-H]^+$
973.674	973.675	PI 44:3	0.59	$C_{53}H_{97}O_{13}P$	[M+H]+
974.362	974.362	PE 36:1	0.09	$C_{41}H_{79}NO_8PIn_2$	$[M+2In-H]^+$
979.476	979.477	PI 36:1	0.34	$C_{45}H_{85}O_{13}P$	$[M+In]^+$
994.334	994.331	PE 38:5	3.11	$C_{43}H_{75}NO_8PIn_2$	$[M+2In-H]^+$
996.345	996.346	PE 38:4	1.98	$C_{43}H_{78}NO_8P$	$[M+2In-H]^+$
999.448	999.445	PI 38:5	2.57	$C_{47}H_{81}O_{13}P$	$[M+In]^+$
1001.458	1001.461	PI 38:4	3.08	$C_{47}H_{83}O_{13}PIn$	$[M+In]^+$
1003.473	1003.477	PI 38:3	3.72	$C_{47}H_{85}O_{13}PIn$	$[M+In]^+$

*:not imaged in dual-polarity MALDI-MSI strategy with a single matrix application step

Table S4 Putative lipids detected by MALDI2-MSI from A549 cells in negative ionization mode.

**Exp. *m/z***	**Cal. *m/z***	**Tentatively identity**	**Delta (ppm)**	**Formula**	**Ion type**
281.2474	281.2486	FA 18:1	4.27	$C_{18}H_{34}O_2$	$[M-H]^-$
283.2627	283.2643	FA 18:0	5.65	$C_{18}H_{36}O_2$	$[M-H]^-$
303.2324	303.233	FA 20:4	1.98	$C_{20}H_{32}O_2$	$[M-H]^-$
408.2171	408.2157	LPC 10:1	3.43	$C_{18}H_{36}NO_7P$	$[M-H]^-$
417.2411	417.2412	LPA O-18:3	0.24	$C_{21}H_{39}O_6P$	$[M-H]^-$
419.257	419.2568	LPA O-18:2	0.48	$C_{21}H_{41}O_6P$	$[M-H]^-$
435.2515	435.2517	LPA 18:1	0.46	$C_{21}H_{41}O_7P$	$[M-H]^-$
450.2595	450.2626	LPE 16:1	6.88	$C_{21}H_{42}NO_7P$	$[M-H]^-$
476.2767	476.2783	LPE 18:2	3.36	$C_{23}H_{44}NO_7P$	$[M-H]^-$
478.2932	478.2939	LPE 18:1	1.46	$C_{23}H_{46}NO_7P$	$[M-H]^-$
480.3087	480.3096	LPE 18:0	1.87	$C_{23}H_{48}NO_7P$	$[M-H]^-$
498.261	498.2626	LPE 20:5	3.21	$C_{25}H_{42}NO_7P$	$[M-H]^-$
500.278	500.2783	LPE 20:4	0.60	$C_{25}H_{44}NO_7P$	$[M-H]^-$
502.2932	502.2939	LPE 20:3*	1.39	$C_{25}H_{46}NO_7P$	$[M-H]^-$
506.2871	506.2888	LPC 16:2;O	3.36	$C_{24}H_{46}NO_8P$	$[M-H]^-$
512.2762	512.2783	LPC 18:5	4.10	$C_{26}H_{44}NO_7P$	$[M-H]^-$
528.2734	528.2732	LPS O-20:5	0.38	$C_{26}H_{44}NO_8P$	$[M-H]^-$
528.3077	528.3096	LPE 22:4*	3.60	$C_{27}H_{48}NO_7P$	$[M-H]^-$
599.3189	599.3202	LPI 18:0	2.17	$C_{27}H_{53}O_{12}P$	$[M-H]^-$
645.4474	645.4501	PA 32:1	4.18	$C_{35}H_{67}O_8P$	$[M-H]^-$
671.4628	671.4657	PA 34:2	4.32	$C_{37}H_{69}O_8P$	$[M-H]^-$
673.4802	673.4814	PA 34:1	1.78	$C_{37}H_{71}O_8P$	$[M-H]^-$
699.495	699.497	PA 36:2	2.86	$C_{39}H_{73}O_8P$	$[M-H]^-$
716.5217	716.5236	PE 34:1	2.65	$C_{39}H_{76}NO_8P$	$[M-H]^-$

718.5392	718.5392	PE 34:0	0.00	$C_{39}H_{78}NO_8P$	$[M-H]^-$
730.5756	730.5756	PE O-36:1*	0.00	$C_{41}H_{82}NO_7P$	$[M-H]^-$
742.5376	742.5392	PE 36:2	2.15	$C_{41}H_{78}NO_8P$	$[M-H]^-$
744.5526	744.5549	PE 36:1	3.09	$C_{41}H_{80}NO_8P$	$[M-H]^-$
760.5132	760.5134	PS 34:1	0.26	$C_{40}H_{76}NO_{10}P$	$[M-H]^-$
764.5202	764.5236	PE 38:5	4.45	$C_{43}H_{76}NO_8P$	$[M-H]^-$
766.5374	766.5392	PE 38:4	2.35	$C_{43}H_{78}NO_8P$	$[M-H]^-$
768.5522	768.5549	PE 38:3	3.51	$C_{43}H_{80}NO_8P$	$[M-H]^-$
770.5685	770.5705	PE 38:2	2.60	$C_{43}H_{82}NO_8P$	$[M-H]^-$
772.5823	772.5862	PE 38:1	5.05	$C_{43}H_{84}NO_8P$	$[M-H]^-$
778.5385	778.5392	CerP 44:6;O_4	0.90	$C_{44}H_{78}NO_8P$	$[M-H]^-$
788.5442	788.5447	PS 36:1	0.63	$C_{42}H_{80}NO_{10}P$	$[M-H]^-$
792.5523	792.5549	Hex2Cer 29:0;O_2*	3.28	$C_{41}H_{79}NO_{13}$	$[M-H]^-$
859.5323	859.5342	PI 36:3	2.21	$C_{45}H_{81}O_{13}P$	$[M-H]^-$
861.5502	861.5499	PI 36:2	0.35	$C_{45}H_{83}O_{13}P$	$[M-H]^-$
863.5643	863.5655	PI 36:1	1.39	$C_{45}H_{85}O_{13}P$	$[M-H]^-$
883.5372	883.5342	PI 38:5	3.40	$C_{47}H_{81}O_{13}P$	$[M-H]^-$
885.5495	885.5499	PI 38:4	0.45	$C_{47}H_{83}O_{13}P$	$[M-H]^-$
887.562	887.5655	PI 38:3	3.94	$C_{47}H_{85}O_{13}P$	$[M-H]^-$
889.579	889.5812	PI 38:2	2.47	$C_{47}H_{87}O_{13}P$	$[M-H]^-$

*:not imaged in dual-polarity MALDI-MSI strategy with a single matrix application step

Matrix deposition device

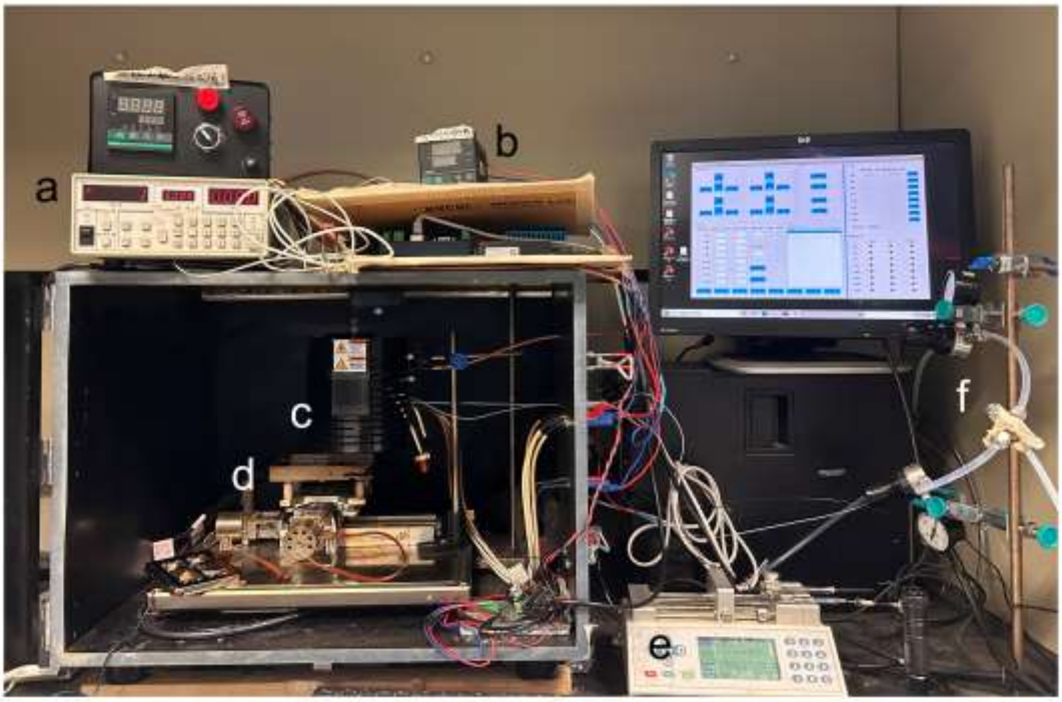

Fig. S1 A schematic diagram of the home-built electron-thermo-spray device. The device consists of several key components: (a) a high-voltage power supply, + 5000 V, (b) a temperature controller 60-80°C, (c) a heating tube and a capillary nozzle (i.d. = 40 μm), (d) a motorized X-Y positioning stage controller, (e) a syringe pump, and (f) a sheath gas supply set at 80 psi.

Lipid profile obtained from tissues and cells

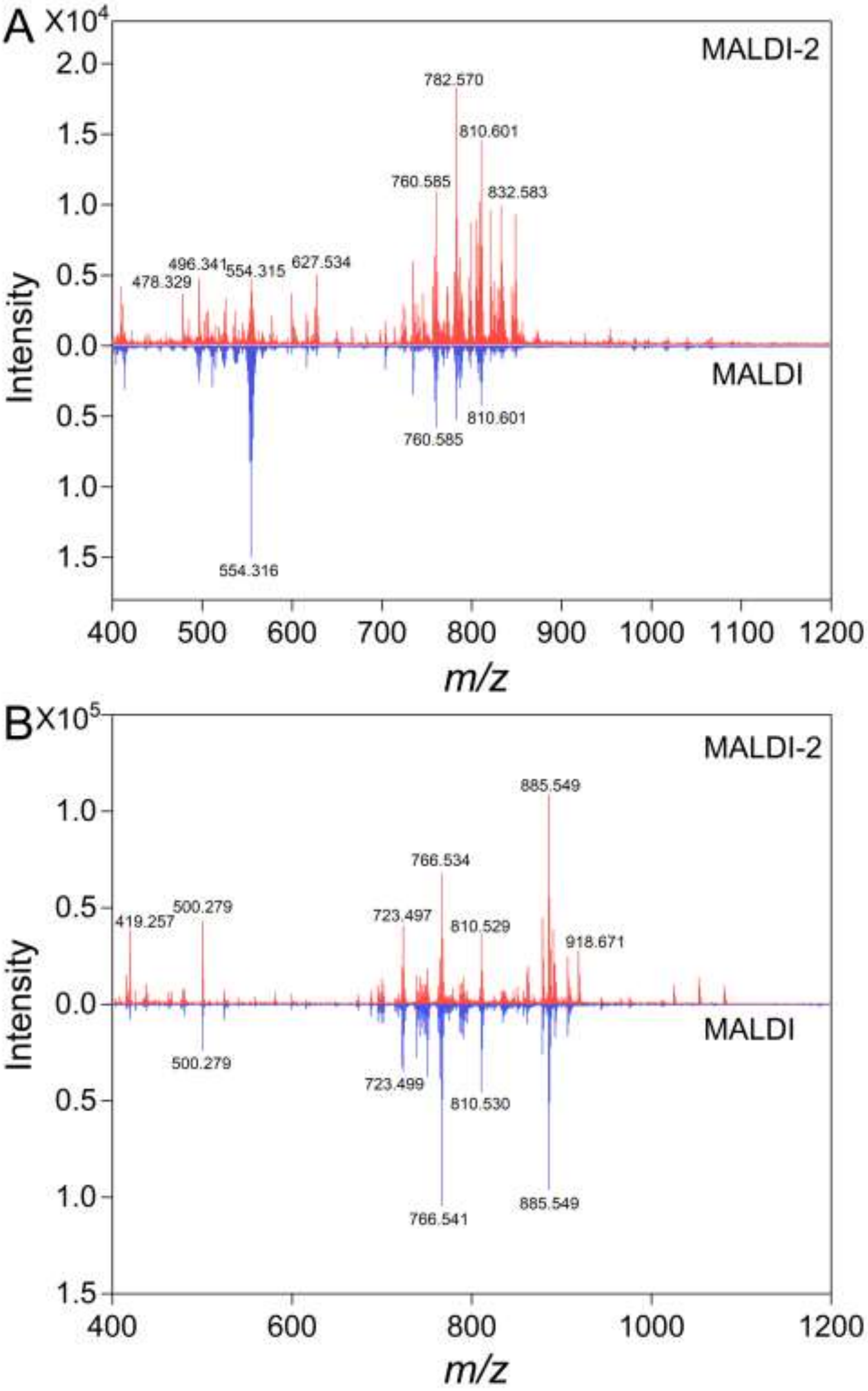

Fig. S2 Average mass spectra obtained from three distinct regions of mouse kidney

sections were analyzed by MALDI-2 (red color) and MALDI (blue color) in (A) positive ionization mode and (B) negative ionization mode using NEDC as the matrix.

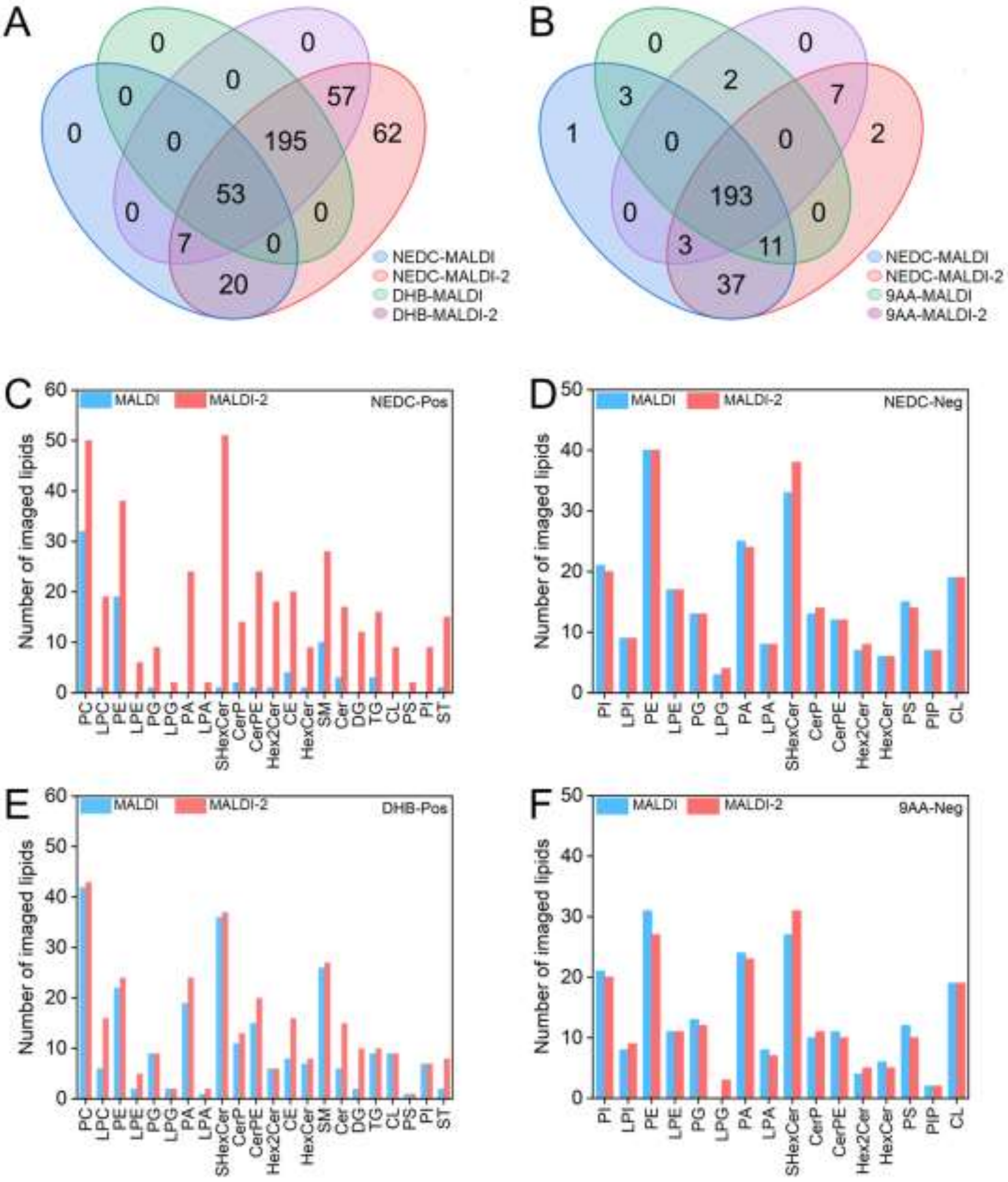

Fig. S3 Comparison of lipid imaging using different matrixes including NEDC, DHB, and 9AA in mouse kidney sections obtained by MALDI- and MALDI-2-MSI. The comparison of imaged lipid number between using DHB and NEDC as matrixes in (A) positive ionization mode, and (B) using NEDC and 9AA as matrixes in the negative ionization mode. The number of imaged lipid species of different lipid classes obtained using NEDC as the matrix in (C) positive ionization mode and (D) negative

ionization mode using MALDI- and MALDI-2-MSI. The number of imaged lipid species of different lipid classes obtained using DHB as the matrix in (E) positive ionization mode and using 9AA as the matrix in (F) negative ionization mode using MALDI- and MALDI-2-MSI.

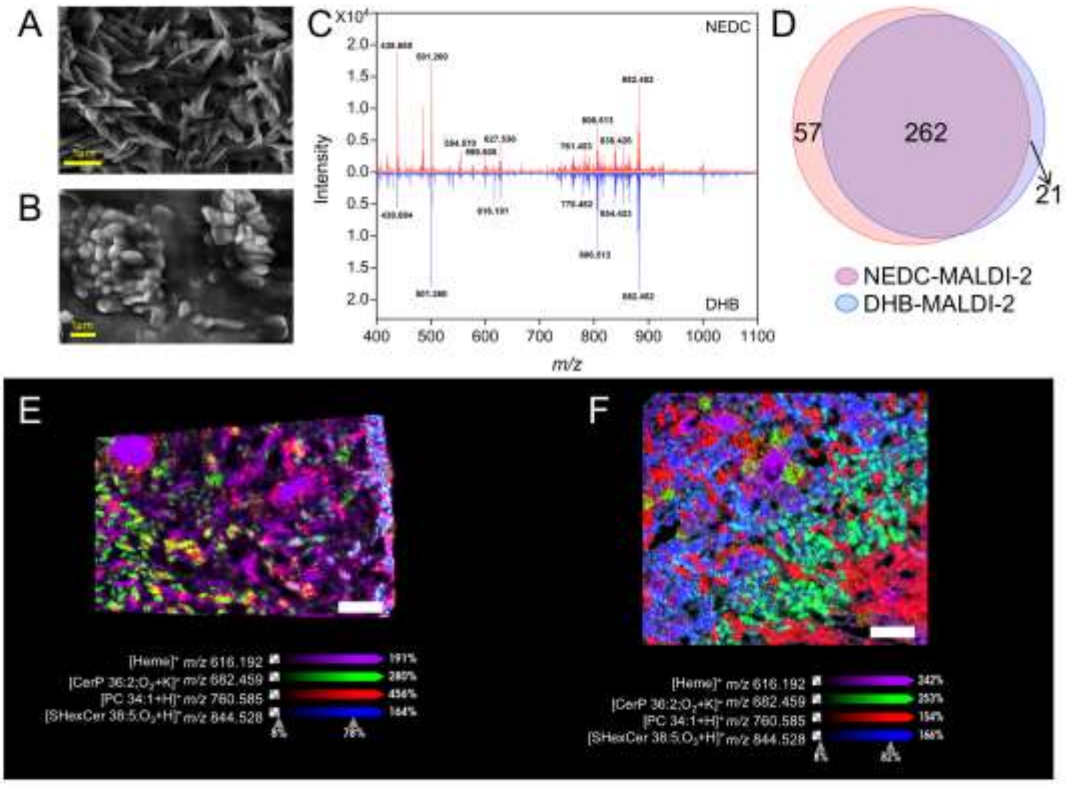

Fig. S4 Comparison of high-resolution imaging ability using DHB and NEDC matrix of MALDI-2-MSI. The crystal size of (A) NEDC and (B) DHB. (C) Mass spectra obtained using NEDC as the matrix (upper, red color) and DHB as the matrix (lower, blue color). (D) The comparison of imaged lipid number between using DHB and NEDC as matrixes with 10 μm resolution in the positive ionization mode. High-resolution RGB MALDI2-MS images of mouse kidney using (E) DHB as the matrix and (F) NEDC as the matrix in positive ionization mode. Scale bar represents 200 μm.

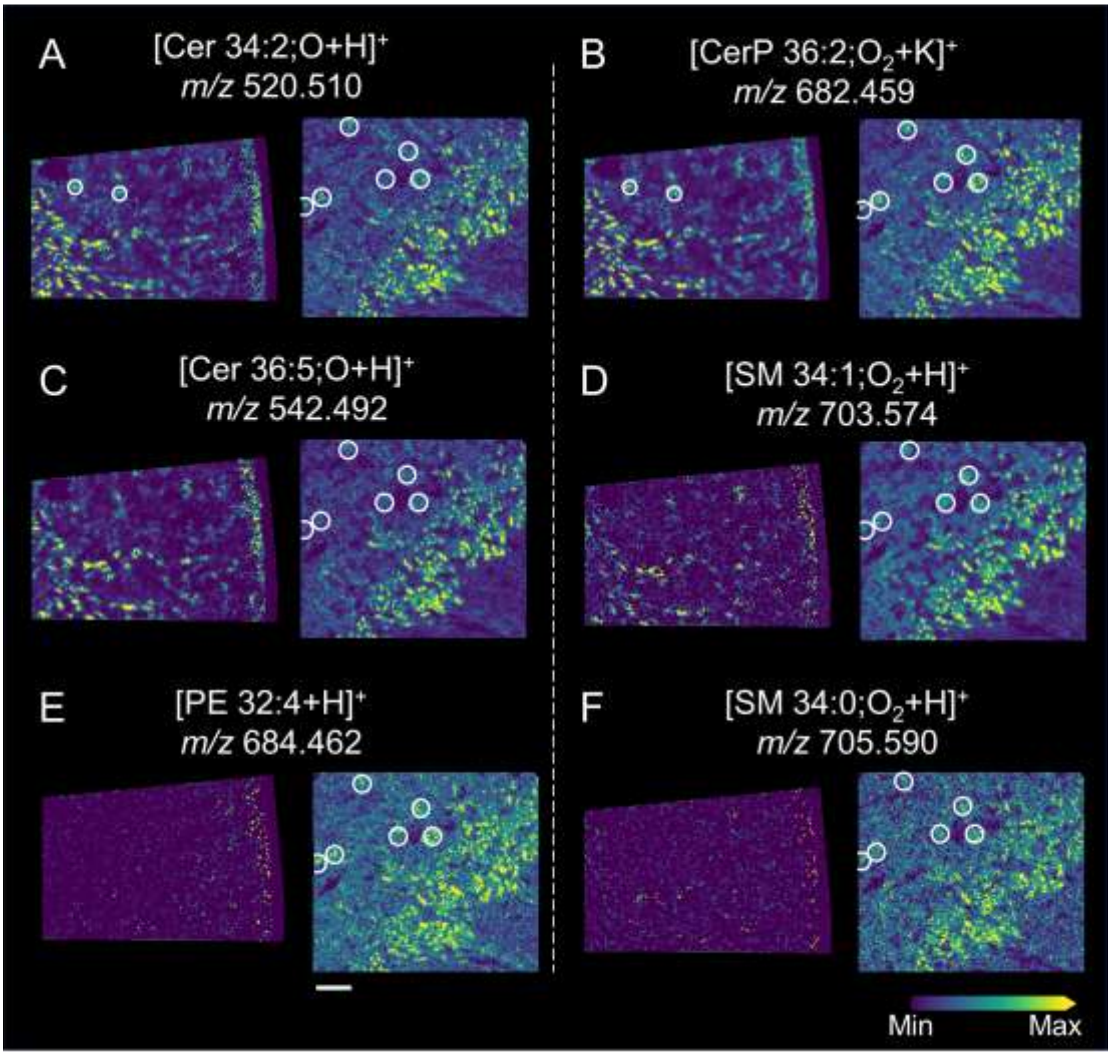

Fig. S5 Comparison of representative high-resolution images using DHB (left) and NEDC (right) matrix of MALDI-2-MSI. MS images of (A) [Cer 34:2;O+H]$^+$, (B) [CerP 36:2;O_2+K]$^+$, (C) [Cer 36:5;O+H]$^+$, (D) [SM 34:1;O_2+H]$^+$, (E) [PE 32:4+H]$^+$, and (F) [SM 34:0;O_2+H]$^+$ in mouse kidney tissue sections. Scale bar represents 200 μm.

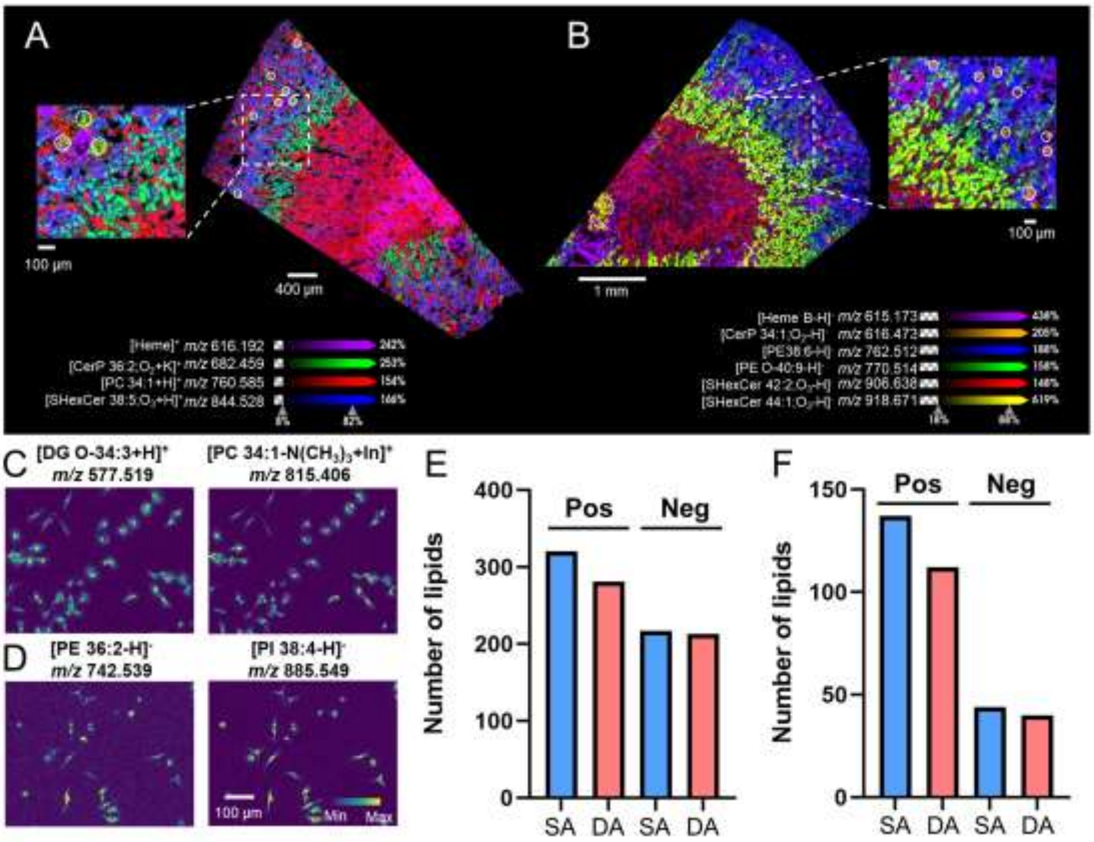

Fig. S6 High resolution images obtained from mouse kidney sections and cells in positive and negative ionization modes. High-resolution RGB MALDI-2-MS images of mouse kidney in (A) positive ionization mode and (B) negative ionization mode using single acquisition (SA). MS images of cells in (C) positive ionization mode and (D) negative ionization mode. The number of lipids detected in positive (Pos) and negative (Neg) ionization modes using SA of MALDI-2 on two slides and double acquisition (DA) with dual-polarity MALDI-MSI strategy of a single matrix application step on the same slide of (E) mouse kidney and (F) A549 cells.

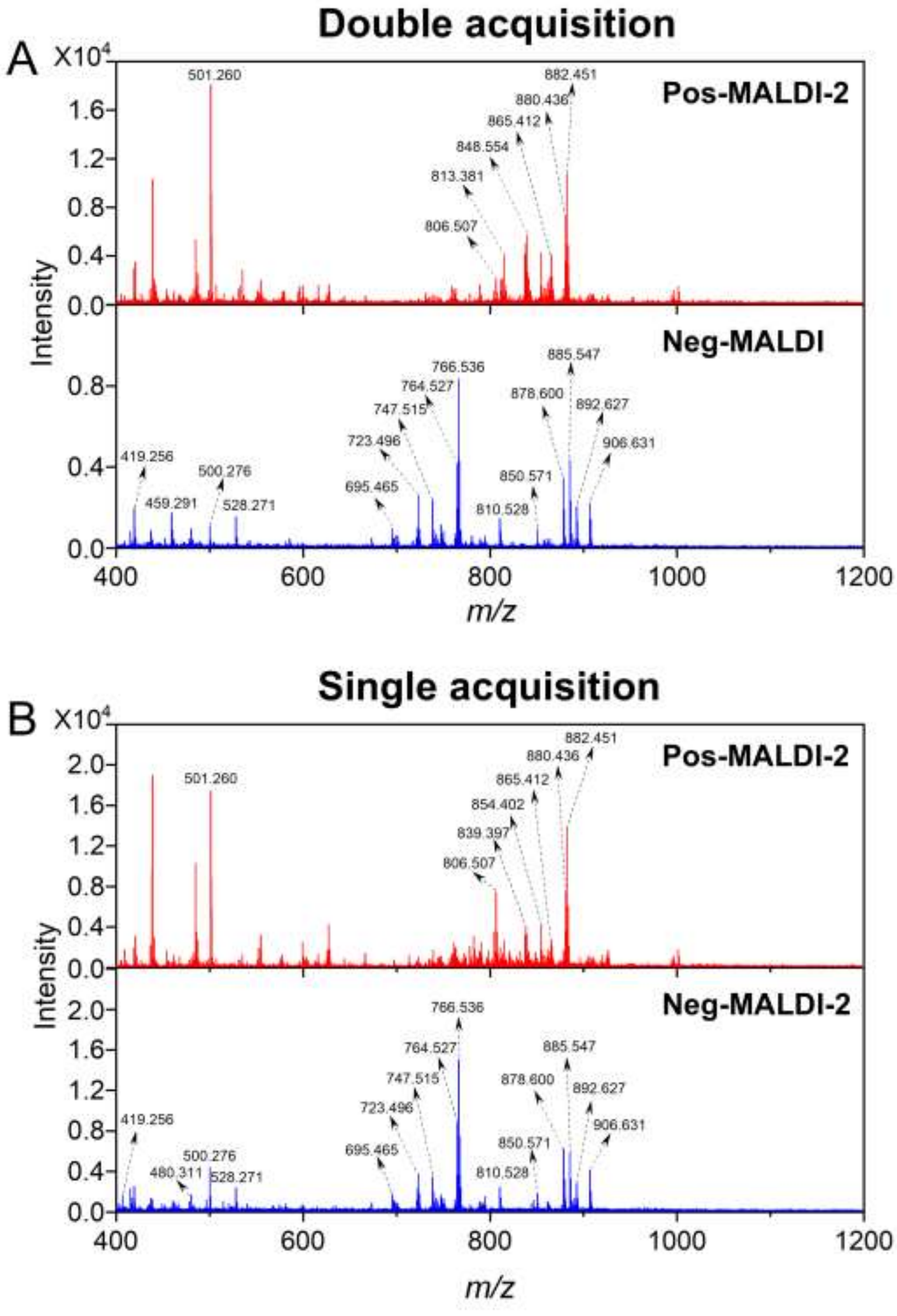

Fig. S7 (A) Mass spectra obtained from the same area of mouse kidney sections were analyzed using MALDI in negative ionization mode (blue color) during the first acquisition and MALDI-2 in positive ionization mode (red color) during the second acquisition. (B) Mass spectra obtained from different area of mouse kidney section

were analyzed using MALDI-2 in both positive (red color) and negative (blue color) ionization modes.

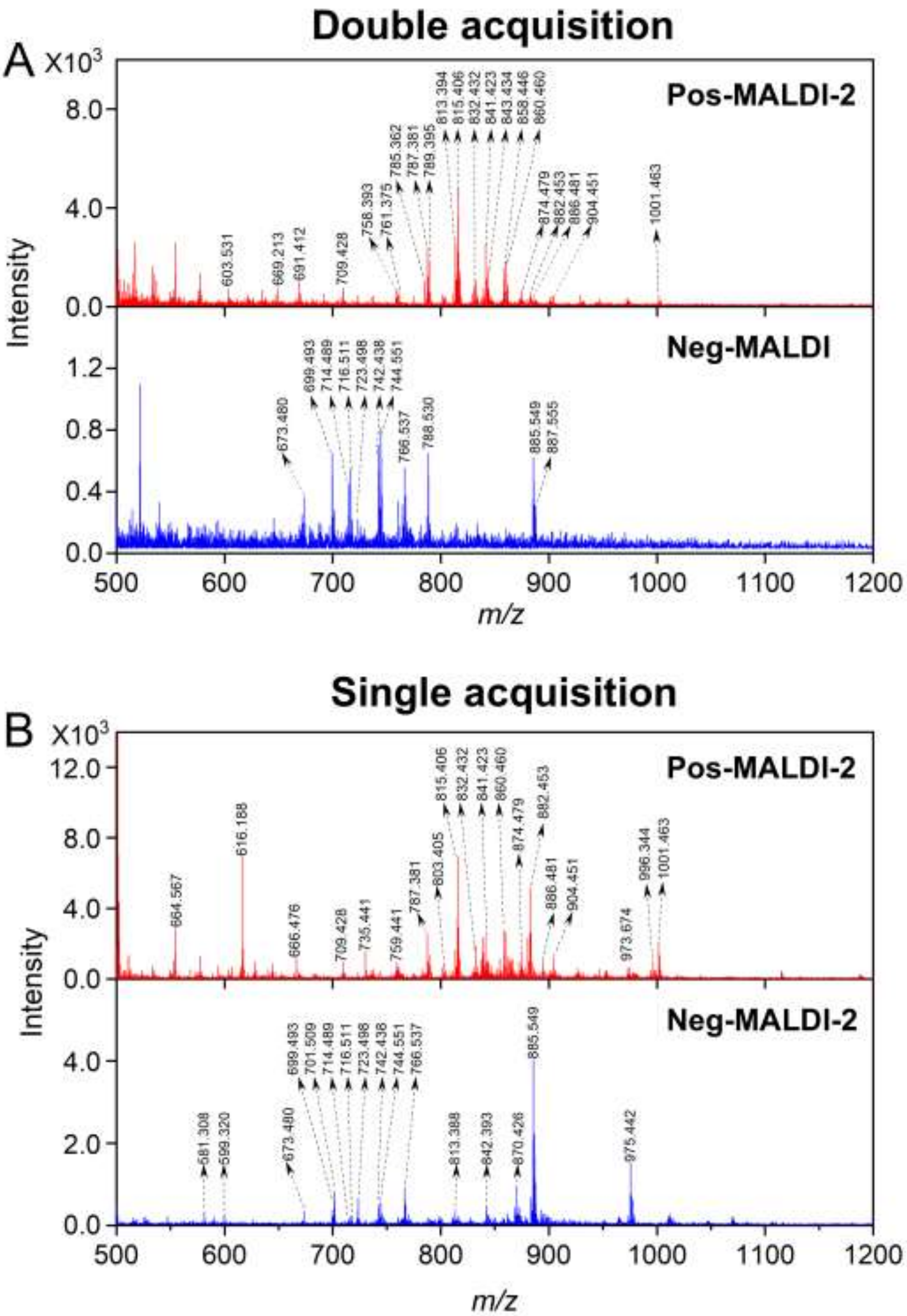

Fig. S8 (A) Mass spectra obtained from the same area of A549 cells were analyzed using MALDI in negative ionization mode (blue color) during the first acquisition and MALDI-2 in positive ionization mode (red color) during the second acquisition. (B) Mass spectra obtained from different area of A549 cells were analyzed using

MALDI-2 in both positive (red color) and negative (blue color) ionization modes.

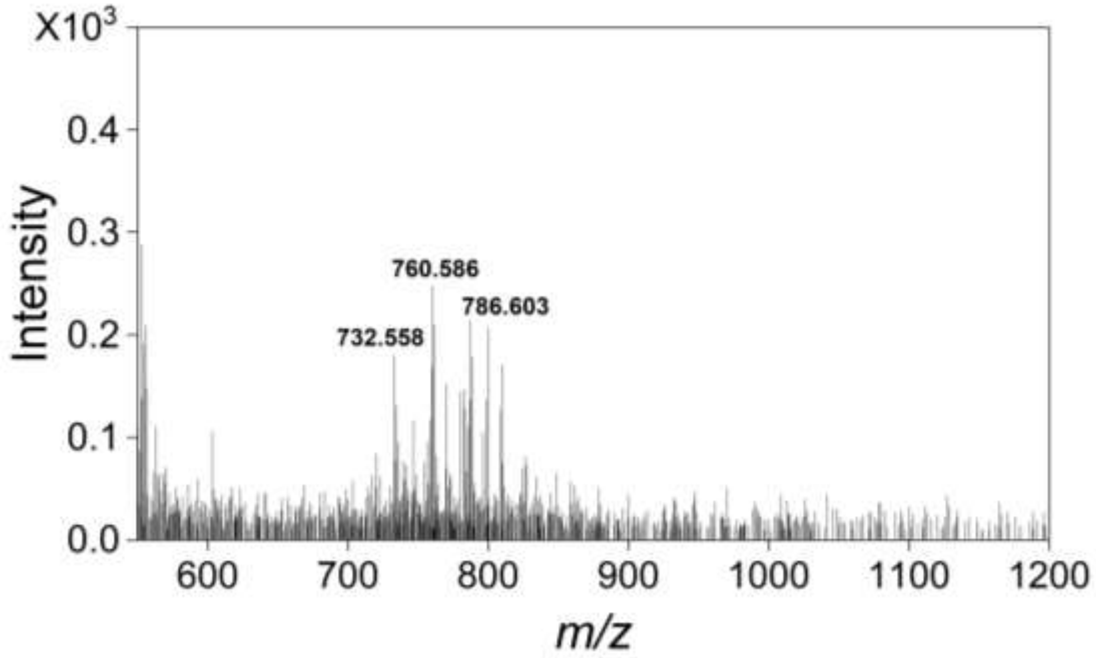

Fig. S9 Mass spectrum of A549 cells obtained in positive ionization mode using NEDC-based MALDI-MSI.

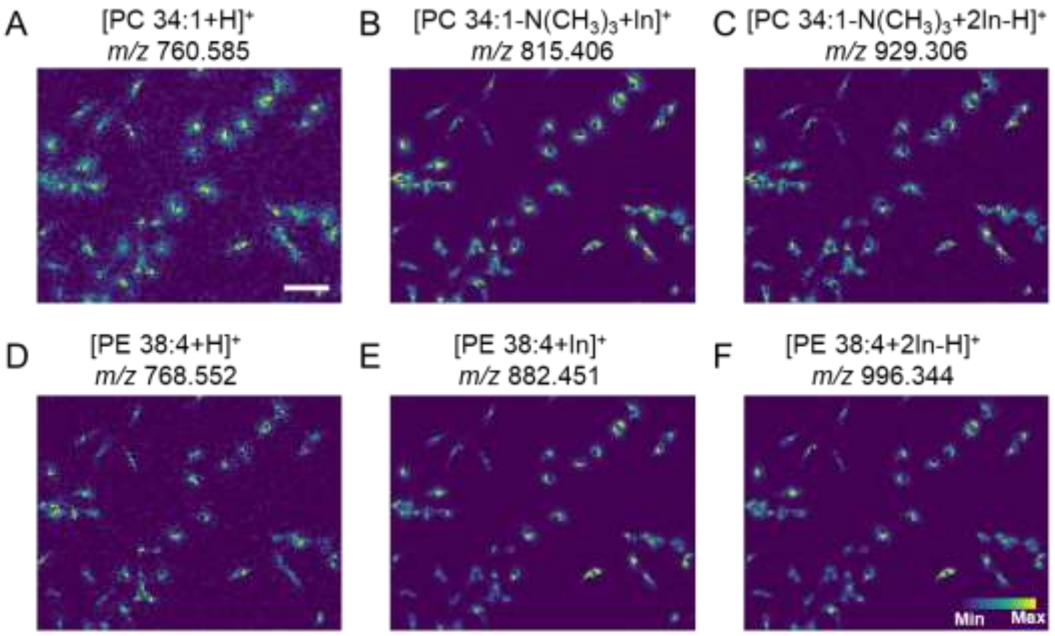

Fig. S10 MS images of lipids with different adducts, including $[M+H]^+$, $[M+In]^+$, and $[M+2In-H]^+$.

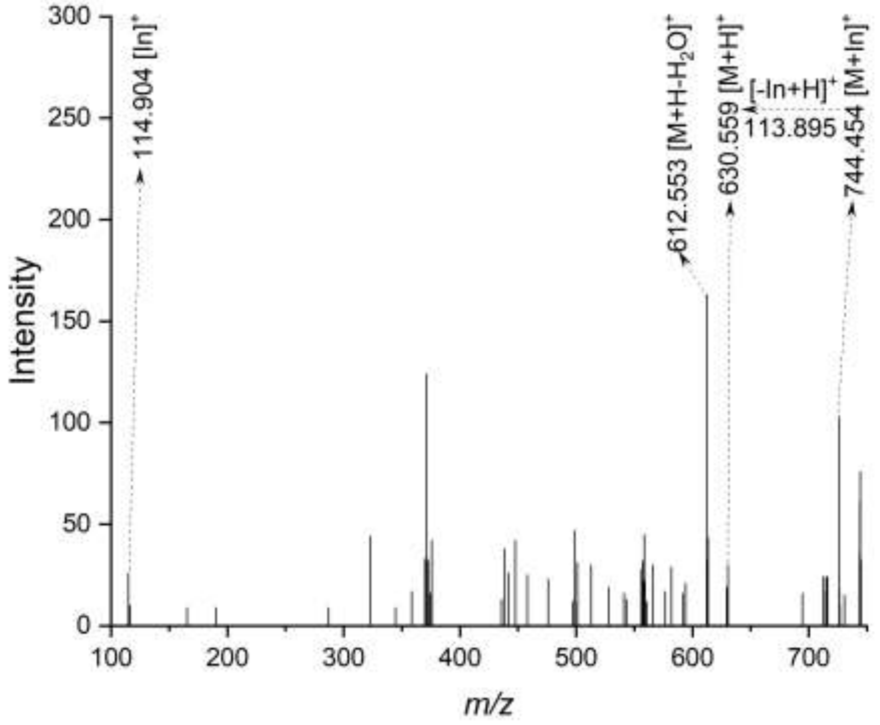

Fig. S11 Example of MS/MS spectrum of $[M+In]^+$.

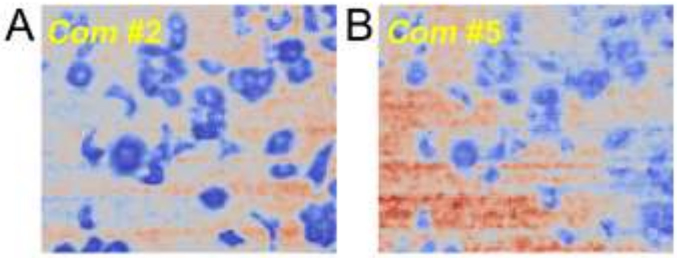

Fig. S12 Lipid-driven in situ PLSA analysis.

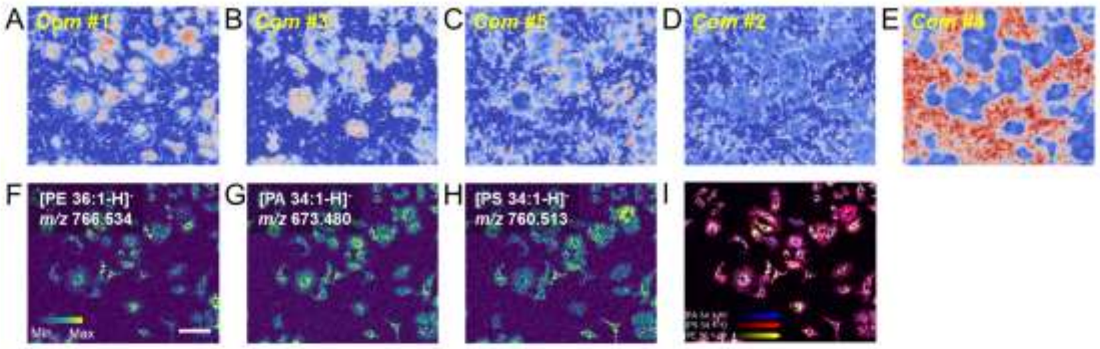

Fig. S13 Spatially resolved lipidomics of same cell slide in negative ionization mode. (A-E) Lipid-driven in situ PLSA analysis. MS images of representative lipids including (F) [PE 36:1-H]$^-$, (G) [PA 34:1-H]$^-$ and (H) [PS 34:1-H]$^-$ and (I) merged image. The scale bar represents 100 μm.
